# Post-crotonylation oxidation by a monooxygenase promotes acetyl-CoA synthetase degradation in *Streptomyces roseosporus*

**DOI:** 10.1038/s42003-023-05633-0

**Published:** 2023-12-08

**Authors:** Bing-Bing Ma, Chen-Fan Sun, Jing-Yi Zhou, Shuai-Lei Gu, Xin-Yi Dai, Yan-Zhen Chen, Qing-Wei Zhao, Xu-Ming Mao

**Affiliations:** 1 Department of Clinical Pharmacy, the First Affiliated Hospital & Institute of Pharmaceutical Biotechnology, School of Medicine, Zhejiang University, 310058 Hangzhou, China; 2 Zhejiang Provincial Key Laboratory for Microbial Biochemistry and Metabolic Engineering, 310058 Hangzhou, China; 3 College of Life Sciences, Zhejiang University, 310058 Hangzhou, China; 4 Zhejiang Provincial Key Laboratory for Drug Evaluation and Clinical Research, 310006 Hangzhou, China

**Keywords:** Bacterial genetics, Bacteriology

## Abstract

Protein post-translational modifications (PTMs) with various acyl groups play central roles in *Streptomyces*. But whether these acyl groups can be further modified, and the influences of these potential modifications on bacterial physiology have not been addressed. Here in *Streptomyces roseosporus* with rich crotonylation, a luciferase monooxygenase LimB is identified to elaborately regulate the crotonylation level, morphological development and antibiotic production by oxidation on the crotonyl groups of an acetyl-CoA synthetase Acs. This chemical modification on crotonylation leads to Acs degradation via the protease ClpP1/2 pathway and lowered intracellular crotonyl-CoA pool. Thus, we show that acyl groups after PTMs can be further modified, herein named post-PTM modification (PPM), and LimB is a PTM modifier to control the substrate protein turnover for cell development of *Streptomyces*. These findings expand our understanding of the complexity of chemical modifications on proteins for physiological regulation, and also suggest that PPM would be widespread.

## Abstract (web summary)

Protein crotonylation, as one of post-translational modifications (PTMs), can be further modified, herein named post-PTM (PPM) by a monooxygenase in *Streptomyces* to regulate substrate protein turnover and cell physiology.

## Introduction

The genus *Streptomyces* is the soil-dwelling bacteria renowned for its plentiful production of antibiotics^1–3^. It also adopts complex life cycle with morphological differentiation from substrate mycelia into aerial mycelia and subsequent spores. More often, it concurrently produces bioactive natural products for bacterial survival advantages. Most studies to understand the regulation of morphological development and bioactive compound production have been focusing on gene expression networks^4,5^, even along with small molecules, such as c-di-GMP, to modulate the transcriptional strength^6,7^. It has been becoming a consensus that post-translational modifications (PTMs) on proteins are emerging as critical mechanisms to regulate both morphological development and natural product biosynthesis in *Streptomyces*^8^.

There are over 400 PTMs have been discovered. It is generally known that PTMs occur with various covalent modifications from small proteins, such as ubiquitin (Ub) and SUMO in eukaryotes^9,10^, and small chemical groups, such as phosphate, methyl and acyl groups, etc^11^. In *Streptomyces*, tagging of a small protein Pup (prokaryotic ubiquitin-like protein) on the Glu residue for proteasome-mediated degradation^12–14^, phosphorylation within two-component systems (TCSs)^15,16^, and an unusual persulfide modification on the Cys residue of AdpA, etc, have been shown to regulate secondary metabolite production and cell differentiation^17^. Moreover, acylations on ε-NH_2_ of Lys, including acetylation^18^, succinylation^19^ and crotonylation^20^, have also been reported to globally regulate cell metabolism. Crotonylation is one of the main PTMs in *S. roseosporus* for over 19% total proteins^20^, while both crotonylation and acetylation regulate antibiotic production^18,21^. Nevertheless, it has been neglected whether there is any new hierarchical regulation beyond but based on PTMs to regulate these physiological processes of *Streptomyces*.

Acylations serve as the most diverse modifications on proteins and 12 types of main acyl-PTMs have been identified to date^22^. But interestingly, the small covalent proteins Ub and SUMO in eukaryotes can be further modified through phosphorylation and acetylation^10,23,24^. However, whether these small acyl groups can be further modified after PTMs (post-PTM modifications (PPMs)) has not been reported. Moreover, most PTMs are reversibly catalyzed by enzymes (writers and erasers), and recognized by interactive proteins (readers) to mediate the regulation^22,25^, as well in *Streptomyces*^19,20^. But whether there are enzymes working as PTM modifiers on acyl groups for PPMs, and the roles of PPMs on physiological regulation of *Streptomyces* remain elusive.

In actinomycetes, acyl-CoAs are not only the donors for acylations, but also building blocks and biosynthetic precursors for backbone assembly and post-line modifications of natural products, and there exist numerous tailoring enzymes conferring structure complexity of these compounds^26,27^. All these provide the basis and simplicity for the genome mining of potential enzymes as PTM modifiers. Conjugative iso-peptidyl and olefinic bonds within a crotonyl group confer the C-C double bond reactive to the nucleophiles^28^. This structural derivation will eliminate the unique π-π rigid and planar configuration of the crotonyl group, and possibly lead to new biological regulation^25^. In fact, an epoxy can be introduced on a conjugative ketone-ene bond, a mimic of crotonylation, by a luciferase-like monooxygenase MsnO8 during the biosynthesis of an anti-tumor natural product mensacarcin in *S. bottropensis* (Fig. S1a)^29^.

Our previous crotonyl-proteomic work in a daptomycin producer *S. roseosporus* has shown that crotonylation is abundant and widespread on key enzymes for various metabolic pathways^20^. Inspired by the above reaction exerted by MsnO8 on a natural product^29^, here in *S. roseosporus*, we identified a luciferase-like monooxygenase LimB as a PTM modifier to catalyze oxidation on the crotonyl groups of an acetyl-CoA synthetase Acs and promote its degradation for the elaborate regulation on morphological development and antibiotic production. This work provides evidence that acyl groups can be further modified after PTM, named PPM (post-PTM modification). This modification is involved in protein turnover and subsequent physiological regulation in *Streptomyces*. This work expands our conceptual understanding of hierarchical chemical modification complexity on proteins, as well as their roles on physiological regulation, and it would be universal in other bacterial systems.

## Results

### A luciferase monooxygenase LimB regulates protein crotonylation of *S. roseosporus*

In an attempt to find potential PTM modifiers on the crotonyl group, we searched the genome of *S. roseosporus* (https://www.ncbi.nlm.nih.gov/assembly/GCA_023716005.1), and 11 proteins annotated as luciferase-like monooxygenases were identified (Table S1). One of these enzymes, Orf6299, herein named LimB with 99.5% identity to LimB from *Streptomyces* sp. PTY08712 (accession number WP_065484148.1), contains an N-terminal domain of flavin-utilizing-monooxygenase as MsnO8 (Fig. S1b).

**Fig. 1:**
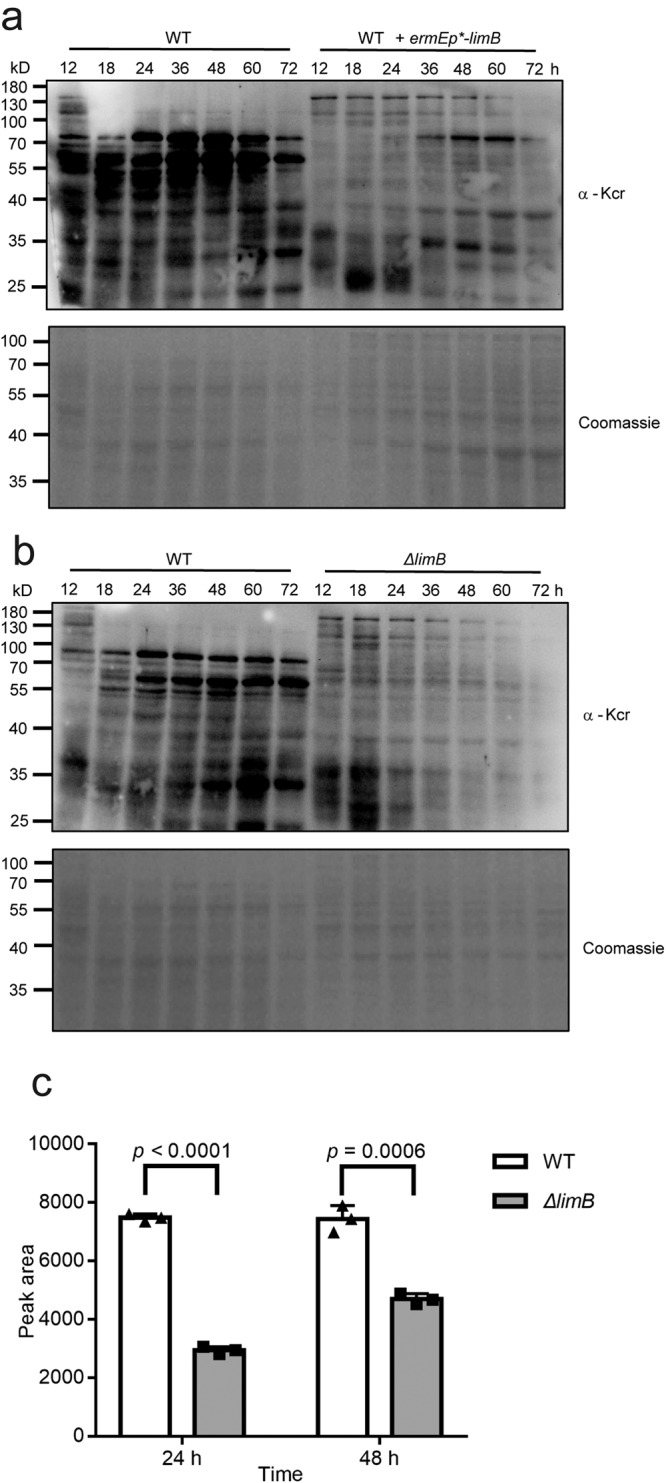
LimB is required for proper protein crotonylaton in *S. roseosporus*. **a**, **b**. Total protein crotonylation was examined in wild type (WT), *limB* over-expression strain (WT + ermEp*-limB) (**a**) and the *limB* null mutant (*ΔlimB*) (**b**). All the strains were fermented in the YEME medium for the time indicated, and the cell lysate was prepared for Western blot with an anti-crotonylation antibody (α-Kcr) or Coomassie blue staining for the loading control. **c** The wild type (WT) and *ΔlimB* strains were cultured in the YEME medium for the time indicated, and the intracellular crotonyl-CoA levels were examined. Statistical significance was determined by Student’s *t* test, *n* = 3. In all panels, error bars show SEM, and *p < 0.05, **p < 0.01, ***p < 0.001, ****p < 0.0001.

We found that overexpression of LimB leads to a drastic decrease in protein crotonylation, especially before 36 h, when cells are under logarithmic growth phase (Fig. S2). Crotonylation in this strain is also much lower in all time points afterwards during the stationary growth phase (Figs. S2 and 1a). This reduced immuno-signal of crotonylation is consistent with the hypothesis that LimB might catalyze oxidation on the crotonyl groups to reduce the apparent total crotonylation signals. Moreover, overexpression of LimB orthologs from other species, including *S. coelicolor* M145, *S. albus* J1074, and *Escherichia coli* DH5α (Fig. S3), consistently reduces crotonylation in *S. roseosporus*, respectively (Fig. S1c), suggesting a conserved function of LimB orthologs to regulate crotonylation. Interestingly, the *limB* in-frame deletion mutant (*ΔlimB*) also shows reduced crotonylation in all time points (Fig. 1b). Meanwhile, we found that this mutant has a significantly lower level of crotonyl-CoA (Fig. 1c), which might account for the reduced crotonylation. All these data suggested that LimB regulates total protein crotonylation of *S. roseosporus*.

### LimB regulates morphological differentiation and antibiotic production of *S. roseosporus*

**Fig. 2:**
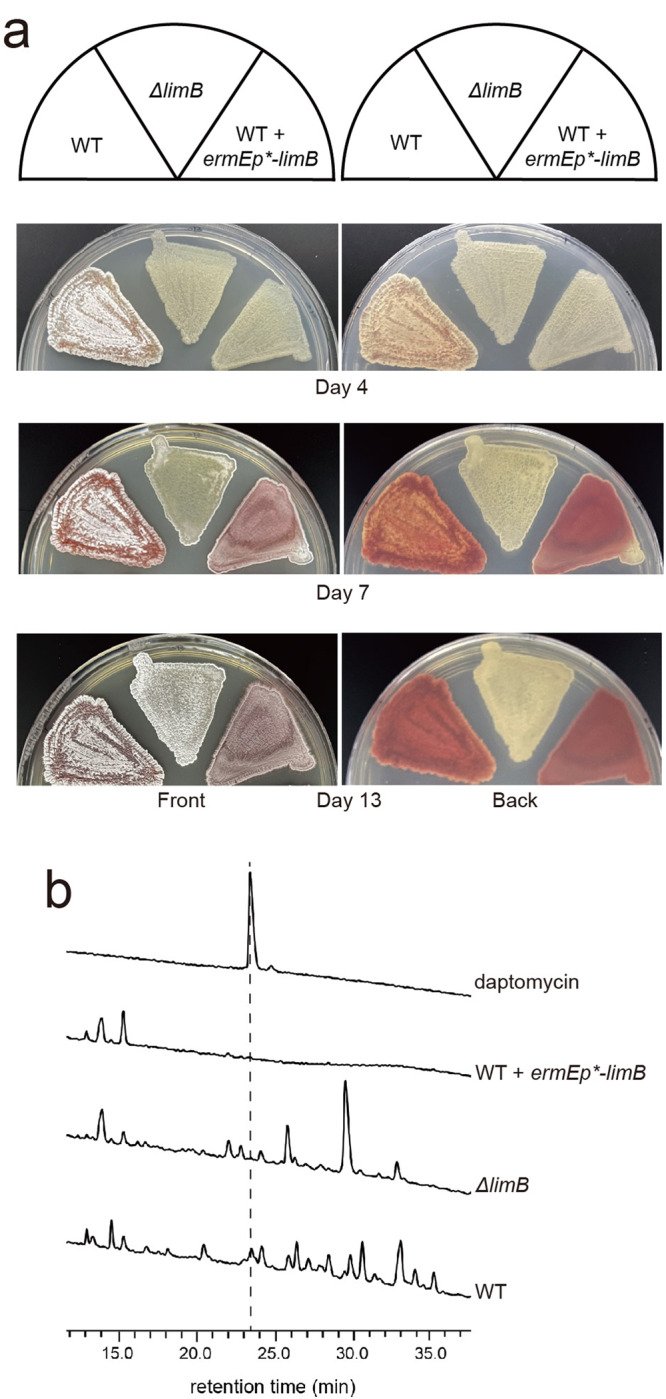
LimB regulates morphological development and antibiotic production of *S. roseosporus*. **a** Phenotype analysis after disturbance of *limB* expression. Three strains as in Fig. 1 were patched on the R5 medium for the days indicated, and the plates were photographed. **b** Antibiotic daptomycin production. The strains as in Fig. 1 were fermented in the YEME medium for 180 h, and the supernatant was subject to HPLC for daptomycin detection. The pure daptomycin was shown as a standard.

Consistent with this abnormal crotonylation levels, we observed delayed morphological development with altered expression of *limB*. On day 4, when the wild type strain (WT) begins to form the white aerial mycelia, both *ΔlimB* and *limB*-overexpression strains grow as substrate mycelia. Moreover, the red pigment, which is produced from a type II polyketide synthase^30,31^, is only produced in WT at this time point. On day 7, when the *limB*-overexpression strain has developed to aerial mycelia and produced the red pigment, no aerial mycelia is observed in the *ΔlimB* mutant yet. Until day 12, the *ΔlimB* mutant produces aerial mycelia, while two other strains have fully sporulated (Fig. 2a). In addition, both the *ΔlimB* and *limB*-overexpression strains lose the capacity to produce the antibiotic daptomycin (Figs. 2b and S4), a representative secondary metabolite from L30^20^. These data suggest that LimB elaborately regulates morphological development and antibiotic production of *Streptomyces*.

### Protein dynamics of LimB during cell development

**Fig. 3:**
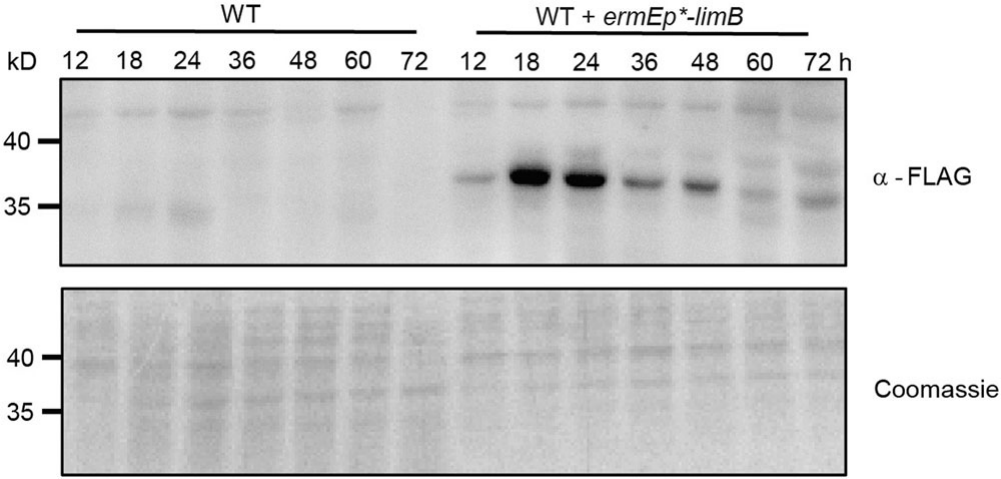
Investigation of LimB protein dynamics in vivo. 3×FLAG-tagged LimB was expressed in WT, and the lysate from the recombinant strain was prepared as in Fig. 1, and subject to Western blot with an anti-FLAG (α-FLAG) antibody or Coomassie blue staining. The data from WT were shown as the control for FLAG-tagged protein expression.

These above phenomena also suggest that the appropriate expression level of LimB might be required to maintain the normal daptomycin production, and prompts us to investigate the LimB protein level during the cell development of *Streptomyces*. We found that LimB protein increases in the logarithmic growth phase (<24 h), but gradually decreases when cells grow into the stationary phase (>24 h), and shows a peak at the phase transition (24 h) (Fig. 3). These data suggest that LimB protein is delicately regulated and are consistent with our observations that disturbance of *limB* expression results in reduced crotonylation. In addition, in a proteasome-deficient mutant with deletion of the protease subunits PrcB/A (*ΔprcB/A*)^20^, LimB protein is much more abundant throughout all the growth phases (Fig. S5), further supporting that LimB protein is regulated through dynamic degradation during cell growth.

### Post-crotonylation oxidation catalyzed by LimB on the acetyl-CoA synthetase Acs

**Fig. 4:**
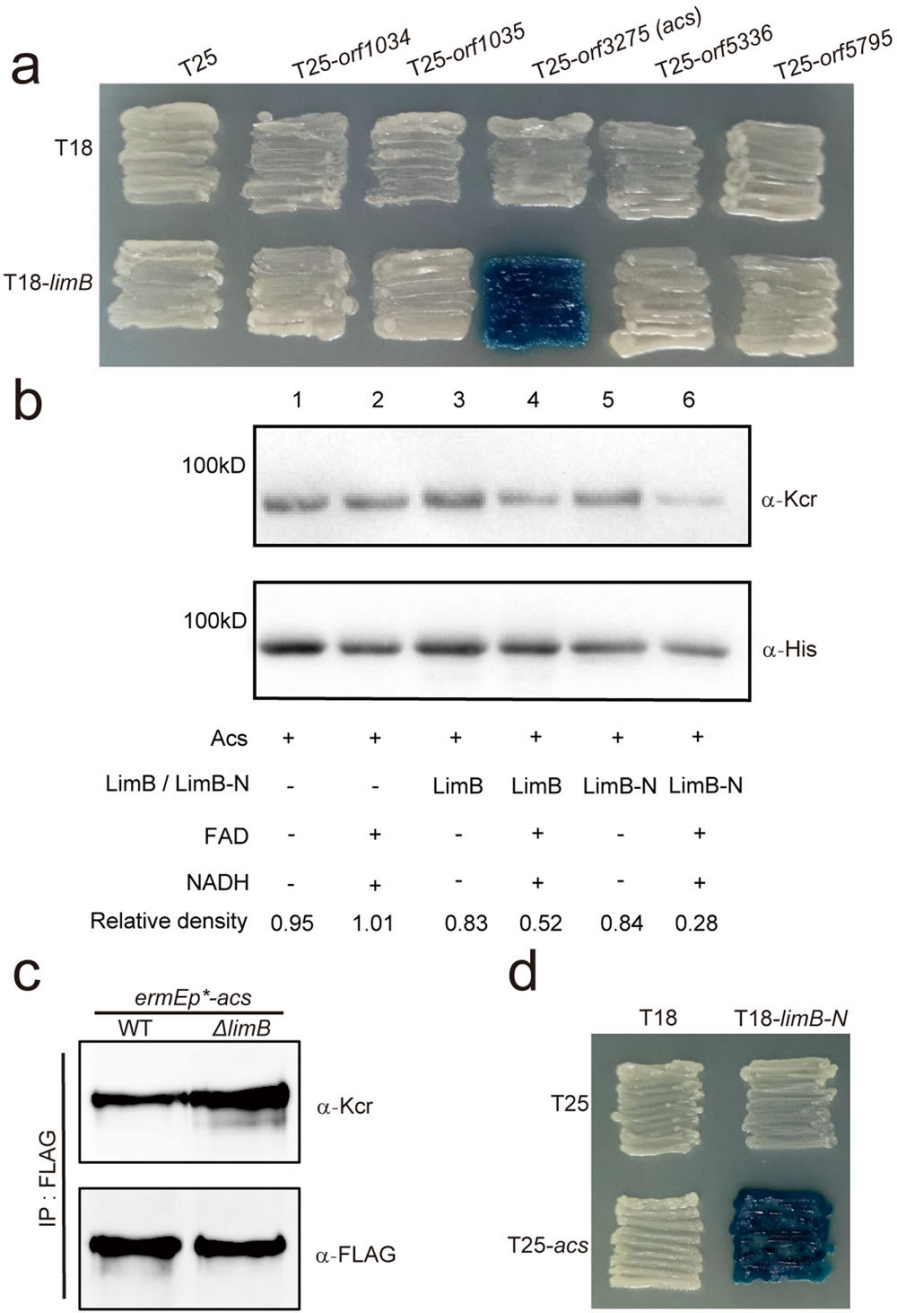
LimB catalyzes post-crotonylation oxidation on Acs. **a**. Bacterial two-hybrid assays for the interactions of LimB and acyl-CoA synthetases. The *E. coli* BTH101 containing LimB (T18-LimB) or the void vector T18 along with other acyl-CoA synthetases (Orf1034, Orf1035, Orf3275, Orf5336, Orf5795) were patched on the LB medium, grown for 3 days and photographed. **b** In vitro oxidation assays with purified His-tagged LimB, LimB-N and Acs. Acs was incubated LimB (lanes 3 and 4) or LimB-N (lanes 5 and 6), coenzymes and Western blot assays were demonstrated with the anti-crotonylation (α-Kcr) antibody for the crotonylation level and the anti-His (α-His) antibody for the loading control of Acs. The control assays without LimB/LimB-N or coenzymes (NADH, FAD) or neither of them were also included (lane 1-3 and 5). **c** In vivo crotonylation assays of Acs after *limB* deletion. Acs was expressed with a 3×FLAG tag in *S. roseosporus*, and immuno-purified from the lysate of WT and the *ΔlimB* mutant. Western blot assays were demonstrated with α-Kcr antibody for the crotonylation level and α-FLAG antibody for the loading control of Acs. **d** Bacterial two-hybrid assays for the interaction of LimB N-terminal domain (LimB-N) and Orf3275 (Acs). The *E. coli* BTH101 containing LimB-N or the void vector T18 along with Acs or the vector T25, respectively, were patched on the LB medium, grown for 3 days and photographed.

Based on the above hypothesis and observations, we assumed that the monooxygenase LimB might be a crotonylation modifier possibly to catalyze oxidation on the crotonyl group. For the proof of this concept, we next tried to identify its crotonylated substrate proteins. Since loss of this monooxygenase leads to reduced crotonyl-CoA production (Fig. 1c), we hypothesized that LimB might positively regulate enzymes involved in acyl-CoA biosynthesis. This *Streptomyces* strain contains two acetyl-CoA synthetases (Orf1035, Orf3275) and three acyl-CoA synthetases (Orf1034, Orf5336, Orf5795), which are all highly crotonylated^20^ (Table S2). Our bacterial two-hybrid assays showed that LimB specifically interacts with acetyl-CoA synthetase Acs (Orf3275) (Fig. 4a). There are six crotonylation sites that have been identified on Acs^20^ (Figs. S6a and S7–S12). In vitro assays showed that LimB reduces the crotonyl-signal of Acs, as compared to the control assays (no LimB, or no coenzymes) (Fig. 4b, lanes 1–4). Furthermore, after immuno-purification of FLAG-tagged Acs from *Streptomyces*, this Acs protein from the *ΔlimB* mutant shows higher crotonylation (Fig. 4c), which supports that LimB negatively regulates Acs crotonylation in vivo, probably through oxidation of the crotonyl group.

Furthermore, we found the N-terminal flavin-utilizing monooxygenase domain (LimB-N) physically interacts with Acs in the bacterial two-hybrid assays (Fig. 4d), and also reduces Acs crotonylation in the in vitro assays with coenzymes FAD and NADH, even much more remarkable than the full length LimB protein (Fig. 4b, lanes 5-6). Moreover, if an Acs variant (Acs^K-Q^) with all six Lys (K) residues mutated to Gln (Q) is used in the above in vitro assays, no significant crotonylation change is observed (Fig. S6b), suggesting that the reduced crotonylation occurs among these six identified residues. All these data suggest that Acs is the substrate of LimB, and LimB reduces Acs crotonylation through oxidation exerted by its N-terminal domain.

### LimB-mediated oxidation promotes Acs degradation

**Fig. 5:**
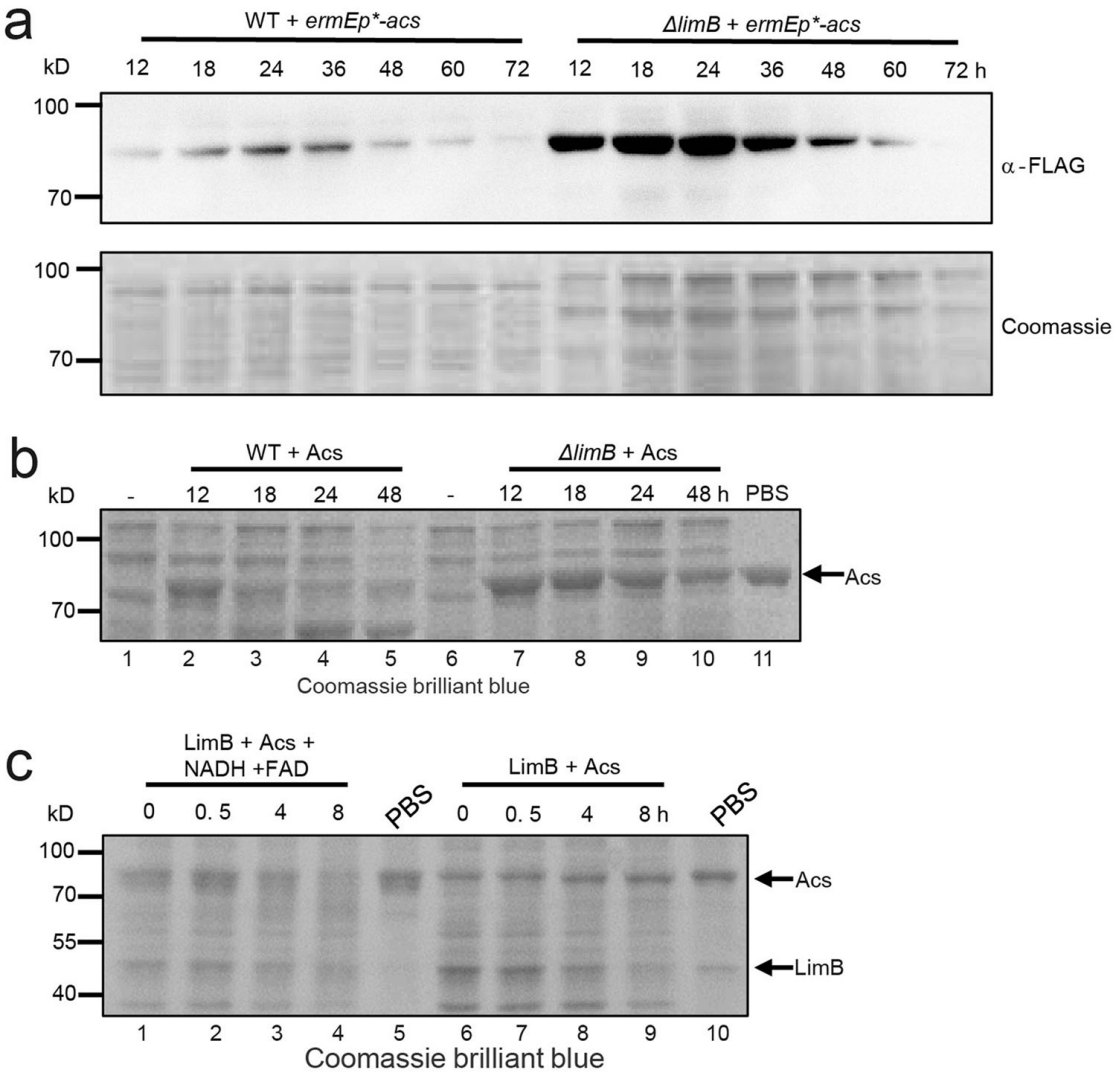
LimB-mediated oxidation promotes Acs degradation. **a** LimB is required for Acs degradation in vivo. 3×FLAG-tagged Acs was expressed in WT and the *ΔlimB* mutant, respectively, and the total cell lysate was prepared and subject to Western blot with an anti-FLAG antibody or Coomassie blue staining as the loading control. **b** LimB is required for Acs degradation in vitro. The purified Acs protein was incubated in the cell lysate of WT and the *ΔlimB* mutant, respectively, which have been fermented for the time indicated. The cell lysate without Acs protein serves as the control for total protein (lanes 1, 6), while Acs in PBS buffer is used as a loading control for the purified protein. The proteins were separated on SDS-PAGE and stained with Coomassie brilliant blue. **c** Acs oxidation is required for its degradation. Acs was initially oxidized by LimB in vitro, or in the same reaction without coenzymes (NADH, FAD) as in Fig. 2b, and subsequently incubated for the time indicated in the cell lysate of the *ΔlimB* mutant (lanes 1–4 and 6–9), which has been fermented for 24 h. Acs in PBS buffer and Acs/LimB in PBS buffer (lanes 5, 10) are used as loading controls for the purified proteins.

Next, the biological consequence of crotonylation oxidation on Acs was investigated. We found that Acs protein also fluctuates during cell growth as LimB. It increases during the logarithmic phase (<24 h), but decreases after 36 h when cells are in the stationary phase (Fig. 5a). However, in the *ΔlimB* mutant, Acs protein stays in higher levels throughout the growth of the microorganism before 60 h, although it still declines rapidly after 60 h of culture (Fig. 5a). These data suggest that LimB negatively regulates Acs stability. To further confirm the requirement of LimB for Acs degradation, Acs is purified from *E. coli* and incubated in the *S. roseosporus* cell lysate prepared from different growth time points. Acs can be apparently degraded in the WT lysate after 18 h, but is fairly stable in the *ΔlimB* mutant lysate in all time points (Fig. 5b), confirming that LimB is required for Acs degradation. Furthermore, if Acs is initially oxidized by LimB in vitro, Acs can be degraded in the *ΔlimB* mutant lysate (Fig. 5c). However, if coenzymes (FAD, NADH) are excluded from the in vitro reactions, Acs’s degradation is precluded even with the purified LimB protein (Fig. 5c). All these data suggest that Acs degradation is dependent on oxidation by LimB.

### Acs is required for morphological development of *S. roseosporus*

**Fig. 6:**
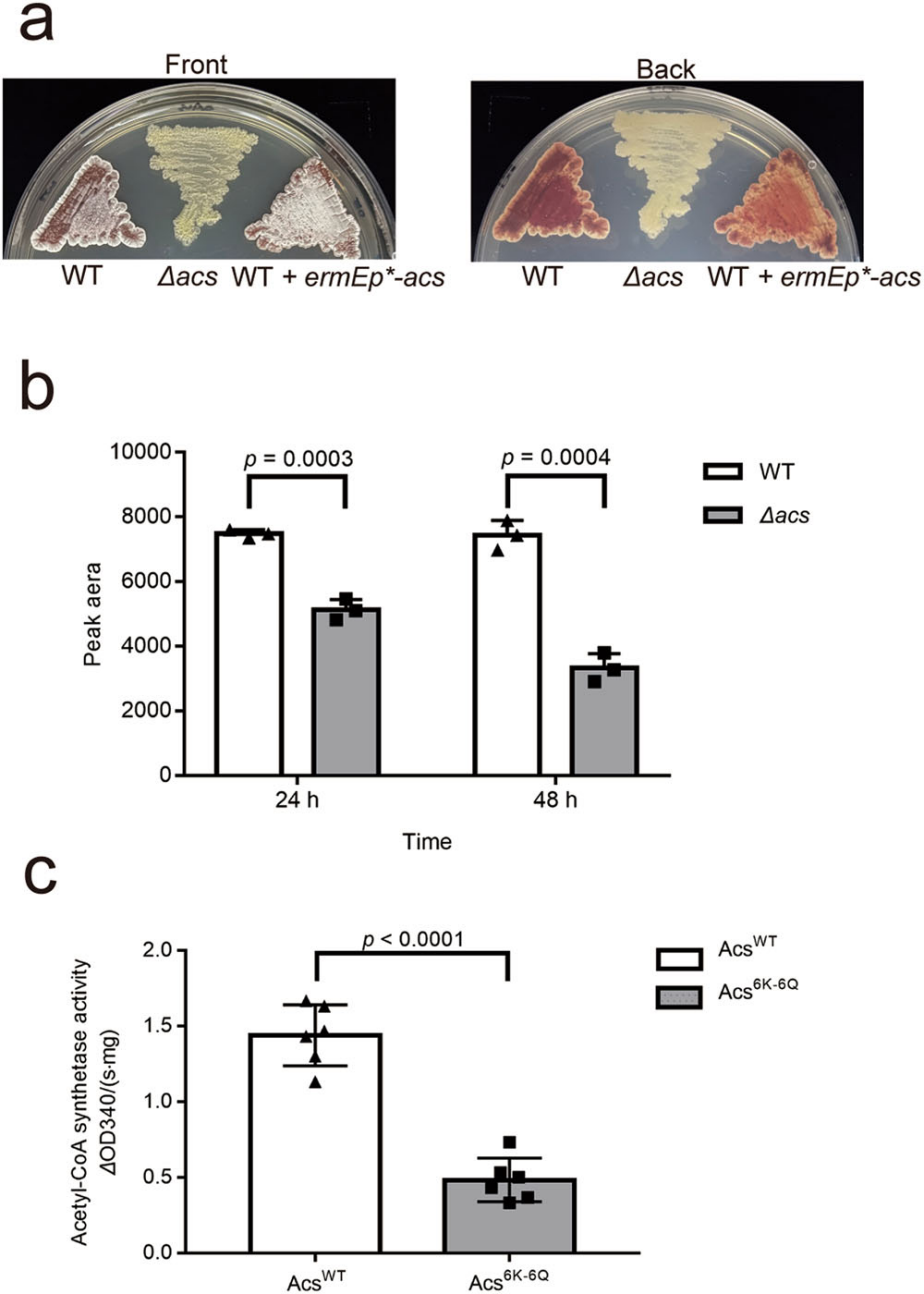
Acs is required for morphological development of *S. roseosporus*. **a** Acs is required for *Streptomyces* morphological development. The wild type (WT), the *Δacs* mutant and the *acs*-overexpression strain (WT + ermEp*-acs) were patched on the R5 medium, incubated for 7 days and photographed. **b** Acs is required for intracellular crotonyl-CoA production. The crotonyl-CoA was examined on HPLC-MS from the cell lysate of WT and the *Δacs* mutant, which have been fermented for 24 and 48 h, respectively. The data were from three independent repeats and the significant difference was presented. Statistical significance was determined by Student’s *t* test, *n* = 3. In all panels error bars show SEM, and *p < 0.05, **p < 0.01, ***p < 0.001, ****p < 0.0001. **c** Crotonylation mimic mutation of Acs abolished its enzymatic activity. Both wild type Acs protein and the Acs variant with six mutations of Lys to Asn were purified from *E. coli*, and their activity of acetyl-CoA synthetase was quantitatively examined. The data were from three independent repeats and significant difference was shown. Statistical significance was determined by Student’s *t* test, *n* = 6. In all panels error bars show SEM, and *p < 0.05, **p < 0.01, ***p < 0.001, ****p < 0.0001.

In line with above data, we found that the *Δacs* mutant is defective in aerial mycelium formation or red pigment production, indicating that Acs is required for morphological development, while the WT and the *acs*-overexpression strains are undisguisable (Fig. 6a). The *Δacs* mutant also shows a lower crotonyl-CoA level than the wild type strain (Fig. 6b), consistent with the reduced crotonylation in the *limB*-overexpression strain. Moreover, we found that the crotonylation mimic variant Acs^6K-6Q^ has lower activity to synthesize acetyl-CoA than the wild type Acs (Fig. 6c), suggesting that this crotonylation is inferior to the Acs catalytic activity. These phenomena can partially explain reduced crotonylation in the *ΔlimB* mutant (Fig. 1b), in which Acs is stable (Fig. 5) but over-crotonylated (Fig. 4c). These data suggest that LimB oxidizes the crotonyl groups of Acs to facilitate its degradation to balance the protein level and intracellular activity of Acs for appropriate acyl-CoA concentration, thus eventually regulating the physiological processes of *Streptomyces*.

### Acs is degraded through the ClpP1/2 protease

**Fig. 7:**
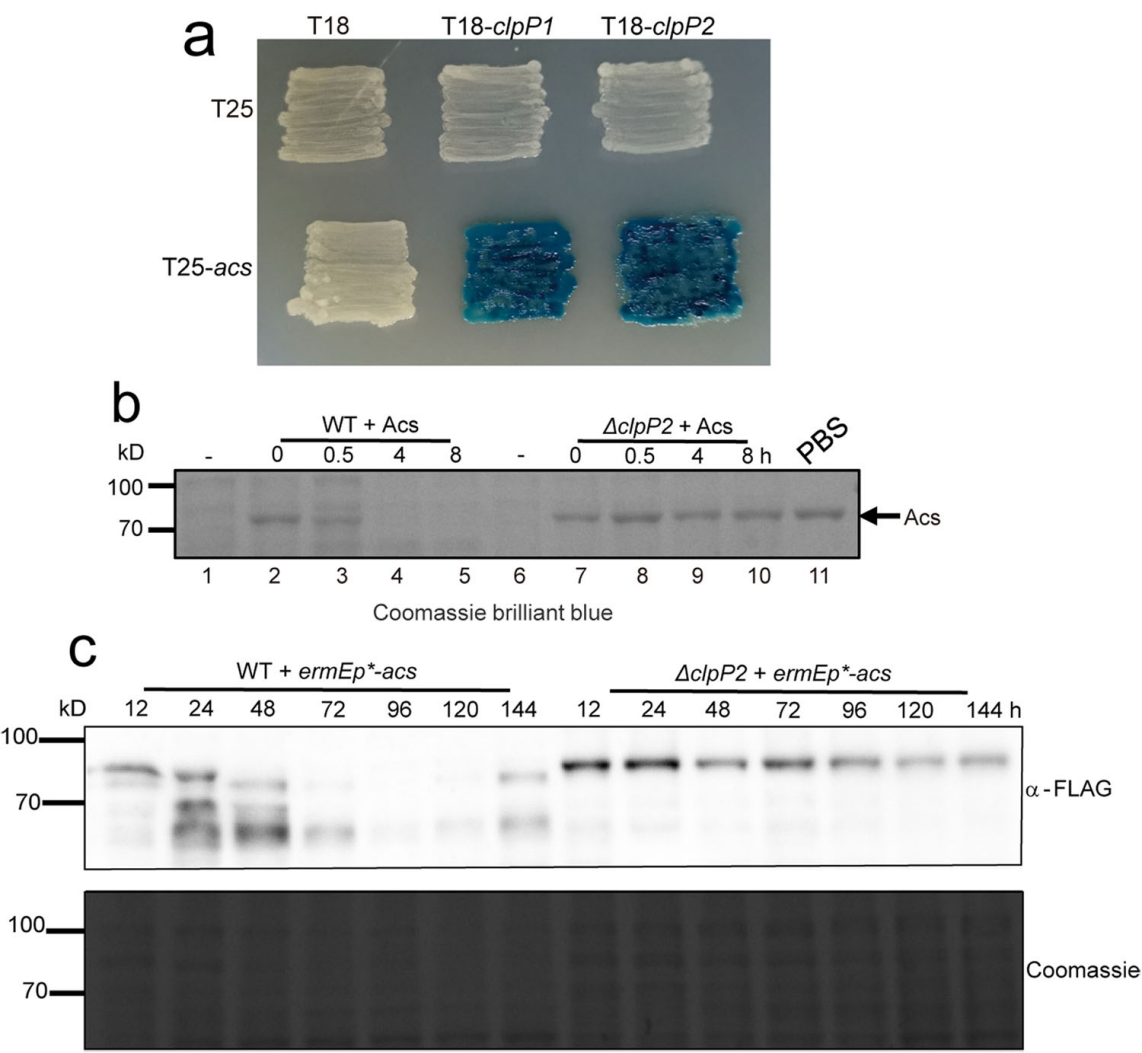
Acs is degraded through the ClpP1/2 protease. **a** Bacterial two-hybrid assays for the interactions of Acs and the protease subunits ClpP1 and ClpP2. The *E. coli* BTH101 containing Acs (T25-Acs) or the void vector T25 along with ClpP subunits were patched on the LB medium, grown for 3 days and photographed. **b** ClpP1/P2 is required for Acs degradation in vitro. The purified Acs protein was incubated in the cell lysate of WT and the *ΔclpP2* mutant, which have been fermented for 24 h in the YEME medium, for the time indicated. The cell lysate without Acs protein serves as the control for total protein, while Acs in PBS buffer is used as a loading control for the purified protein. The proteins were separated on SDS-PAGE and stained with Coomassie brilliant blue. **c** Protease ClpP1/P2 is required for Acs degradation in vivo. 3×FLAG-tagged Acs was expressed in WT and the *ΔclpP2* mutant, respectively, and the total cell lysate was prepared and subject to Western blot with an anti-FLAG antibody or Coomassie blue staining as the loading control.

To further understand Acs turnover regulated by LimB, we next tried to identify the proteases responsible for Acs degradation. In *Streptomyces*, several proteases, including ClpP1/2, putative Clp proteases (Table S3), FstH, Lon, and proteasome (PrcA/B are the α/β subunits and Pup is the tagging peptide)^32–35^, have been shown involved in protein degradation. Our bacterial two-hybrid assays showed that Acs interacts with protease subunits ClpP1 and ClpP2 (Fig. 7a), but not with other protease components (Fig. S13). Consistent with this interaction, we found that the purified Acs protein is degraded rapidly in the wild type lysate, but fairly stable in the *ΔclpP2* mutant lysate (Fig. 7b). Our in vivo data also showed that Acs protein level is higher in the *ΔclpP2* mutant, especially after 36 h (Fig. 7c). All these data suggest that Acs is degraded by the ClpP1/2 protease.

## Discussion

Though many types of acylations have been identified on different residues^22^, there is no report of modifications on the side chains of these acylations. Among these PTMs, 2-hydroxyisobutyrylation is a hydroxyl form of acylation, but this PTM is considered to occur by enzymatic transferring of a 2-hydroxyisobutyryl group to Lys^36,37^. Crotonylation has a reactive C-C double bond. Here we identify a luciferase-like monooxygenase LimB, which proposedly catalyzes oxidation of the side chain of the crotonyl group for post-PTM modification (PPM). Acyl-transferases and deacylases are PTM writers and erasers, respectively, to catalyze the formation and hydrolysis of iso-peptidyl bonds between acyl groups and ε-NH_2_, respectively. Here we show that LimB functions as a crotonylation modifier, in addition to our previously reported Kct1 and CobB as the crotonylation writer and eraser, respectively^20^. PTM writer/eraser partners reversibly catalyze acylations on proteins, while PPM here would be irreversible.

Though post-translational modifications (PTMs) on proteins have been extensively studied in eukaryotes, few reports have revealed that there are several types of PTMs in *Streptomyces* comprehensively regulating cell physiology^17–21^. In particular, acylations widely distribute on enzymes for metabolism and secondary metabolite biosynthesis, and on regulators for secondary metabolism development. Here we also identify that crotonylated Acs is the substrate of LimB, thus establishing the enzymatic reaction to prove the concept of PPM. In addition to crotonylation on Acs here, acetylation of *Streptomyces* Acs has also been reported^38^, and both PTMs impair the enzymatic activity of Acs.

Here we also show that the biological consequence of PPM on Acs is to promote its degradation, and elaborately regulate cell physiology of *Streptomyces*, probably through modulating the intracellular acyl-CoA pool, since the *Δacs* mutant has a lowered crotonyl-CoA level. Based on these observations, the delayed morphological development in both *ΔlimB* mutant and *limB*-overexpression strain would at least result from Acs over-crotonylation and Acs degradation after PPM, respectively, both of which lead to reduced acetyl-CoA synthetase activity. Thus, LimB protein level is exquisitely controlled, as shown in our work, to balance Acs PTM and PPM to appropriately regulate the acetyl-CoA synthetase activity of Acs to eventually affect cell physiology. However, more substrates of LimB should be identified to more comprehensively understand the regulatory mechanism of LimB from the point of view of PPM.

Here we further show that overexpression of some LimB orthologs from other bacteria also reduce crotonylation, suggesting that this monooxygenase-mediated PPM should be widespread in other bacterial systems. Moreover, given the wide distribution of crotonylation even in eukaryotes, it can be expected that this enzymatic post-crotonylation modification would be universal. Since the small protein-mediated PTMs (ubiquitination, sumolyation) can be further modified through phosphorylation and acetylation^10,23^, it is possible that this PPM can also dramatically trigger the signaling consequences like modifications on Ub/SUMO. Our work expands the complexity of protein chemical modifications based on traditional PTMs, and provides a new hierarchical understanding of physiological regulation.

## Methods

### Bacterial strains and growth conditions

All strains listed in this study are shown in Table S4. *Streptomyces* mycelia were cultured in liquid tryptic soy broth (TSB) supplemented with 5% PEG6000, while daptomycin production was carried out in YEME medium containing 4% glucose, 0.5% tryptone, 0.3% yeast extract, and 0.3% malt extract for further cultivation. To promote the synthesis of daptomycin, 0.1% decanoic acid was added to YEME medium every 12 h, starting at 72 h. *Streptomyces* strains were incubated under shaking conditions of 30 °C and 250 rpm. Sporulation of *S. roseosporus* was facilitated using the R5 solid medium, and conjugation was carried out on the MS solid medium. *E. coli* strains were grown in either Luria-Bertani (LB) medium or Terrific Broth (TB) medium with appropriate antibiotics.

### Plasmid construction

All plasmids and primers are provided in Tables S5 and S6, respectively. The fragments encoding *limB* and *limB-N* were cloned into pET32a digested with *Eco*RI*/Bam*HI with primers 31 + 32 and 33 + 34, respectively. The *acs* fragment (primers 23 + 24) and the mutant *acs*^*6K-6Q*^ fragment (primers 25-30) were amplified and inserted into the *Nde*I*/Hin*dIII site of pET28a to get plasmids pET28a-*acs* and pET28a-*acs*^*6K-6Q*^, respectively.

The fragments containing *limB* (primers 35 + 36), *limB-N* (primers 37 + 38), 15 proteases and proteasome genes (primers 49–78) were recombined into the *Kpn*I*/Hin*dIII site of pUT18 for the Bacterial Two-Hybrid System. The five acyl- and acetyl-CoA synthetase genes of *orf1034*, *orf1035*, *orf3275*, *orf5336* and *orf5795* (primers 39-48) were recombined to pKT25 double digested with *Xba*I*/Kpn*I.

All fragments were amplified using corresponding primers (13-22) and cloned into the *Xba*I*/Bg*lII site of pSN7. Phanta Max Super-Fidelity DNA Polymerase (Vazyme biotech Co.Ltd) was used to amplify all fragments with *S. roseosporus* L30, *S. coelicolor* M145, *S. albus* J1074 and *E. coli* DH5α genomic DNA as the template. The cloning process was carried out with the ClonExpress II One Step Cloning Kit (Vazyme biotech Co.Ltd). *E. coli* DH5α (Novagen) was the host for plasmid construction, and conjugation was carried out with the *E. coli* strain ET12567/pUZ8002 to introduce DNA from *E. coli* to *S. roseosporus*.

### Gene knock-out for strain development

To construct pKC1139-based deletion plasmids, we amplified the left and right homologous fragments of *limB*, *acs*, and *clpP2* using primer pairs 1 + 2 and 3 + 4, 5 + 6 and 7 + 8, and 9 + 10 and 11 + 12, respectively. Fragments were sequentially reconstituted into pKC1139 digested with *Xba*I/*Eco*RI by the ClonExpress MultiS One Step Cloning Kit (Vazyme biotech Co. Ltd). Double cross-over homologous recombination was employed to delete *limB, acs*, or *clpP2* via the plasmids pkC1139-*ΔlimB*, pkC1139-*Δacs*, or pkC1139-*ΔclpP2*, respectively, using an in-frame deletion strategy in *S. roseosporus* (Figs. S14–S16).

### Quantification of intracellular crotonyl-CoA concentration

The mycelia were resuspended in PBS buffer after centrifugation at 10,000 rpm at 4 °C for 5 min to remove debris. Next, the protein was precipitated by adding cold 20% TCA and incubating at −20 °C for 2 h. The supernatant was collected after another round of centrifugation as described above. To purify crotonyl-CoA, an Oasis PRiME HLB 96-well Plate (Waters) was used in accordance with the manufacturer’s instructions. The effluent was concentrated using the CentriVap vacuum (Labconco). Then, the samples were performed in Liquid Chromatography-Mass Spectrometry (LC-MS) at a flow rate of 0.5 ml/min using an Agilent Eclipse TC-C18 column (5 μm, 4.6 × 250 mm, Agilent Technologies). The mobile phase was composed of solution A (water containing 20 mM ammonium acetate, pH = 7.4) and solution B (methanol with 20 mM ammonium acetate), with ultraviolet detection set at 254 nm. The High-Performance Liquid Chromatography (HPLC) conditions were set as follows: 0 min, A:B = 75:25%; 20 min, A:B = 0:100%; 25 min, A:B = 0:100%; and 30 min, A:B = 75:25%. The mass spectrometry system was operated in a positive ion mode with an electrospray ionization source^13^.

### Western blot

The strains were cultured in TSB medium for 36 h, and then transferred to YEME medium. The mycelia were collected after a specific period of fermentation and washed three times with PBS (pH 7.4). Ultrasonic fragmentation was then performed using PBS, followed by centrifugation to collect the supernatant. The protein concentration was determined with a Bradford kit (Sangon Biotech) and a microplate reader. 20 μg of protein was then loaded onto each protein gel lane. Electrophoresis was performed at 90 V for 35 min, followed by an increase to 120 V for 1 h. Subsequently, the separated protein bands were visualized using Coomassie brilliant blue staining solution for 1 h. A decolorizing solution containing 60% deionized water, 30% methanol, and 10% glacial acetic acid was used to clean the protein gel. Additionally, the separated proteins were transferred from the gel to a nitrocellulose membrane. The membrane was blocked with a solution containing 5% skim milk for 1 h at room temperature, washed with TBST, and incubated with primary antibodies including anti-FLAG (1:2000), anti-Kcr (1:1000), or anti-His (1:2000). A goat anti-mouse HRP conjugated antibody (1:5000), as the secondary antibody, was used to detect the immunoreactive bands. The protein bands were visualized using a chemiluminescence developer.

### Daptomycin production and analysis

1 ml of the fermentation broth was collected and terminated by adding an equal volume of methanol. The supernatant was harvested by centrifugation and analyzed by HPLC using a Shimadzu LC-20AP with an Agilent ZORBAX SB-C18 column (4.6 × 150 mm, 5 µm) (Agilent Technologies). The HPLC conditions began with 10% acetonitrile for 5 min, then a linear gradient from 10% to 35% acetonitrile over 50 min, and subsequently 45% acetonitrile for 5 min. Next, the condition was switched to 100% acetonitrile for 8 min, and then restored to the initial composition of 10% acetonitrile for another 8 min. A constant flow rate of 1 ml/min was maintained. Detection of the analyte was performed at 215 nm with pure daptomycin as a standard.

For high-resolution liquid chromatography mass spectrometry (HR-LC-MS) analysis of daptomycin, the supernatant was detected on AB Sciex ZenoTOF 7600 with an Agilent Eclipse TC-C18 column (5 μm, 4.6 × 250 mm, Agilent Technologies). The mass spectrometry system was equipped with an electrospray ionization source operating in positive ion mode.

### Bacterial two-hybrid assays

A set of five putative acyl- and acetyl-CoA synthetase genes were cloned into pKT25, and *limB* was recombined into pUT18. Fifteen genes encoding proteases and the proteasome were also inserted into pUT18, and *acs* was inserted into pKT25. Then, the double plasmids (pUT18-gene + pKT25-gene) were transformed into *E. coli* BTH101, which were then grown in LB medium containing 100 μg/ml ampicillin, 50 μg/ml kanamycin, 15 mM IPTG, and 20 μg/ml X-gal at 30 °C for 3 days. A negative control was established using double void plasmids transformed into *E. coli* BTH101.

### Determination of dry weight

The strains were cultured in TSB seed medium for 36 h and then transferred to the YEME medium. Mycelia were then collected at different time points and placed in a metal bath at 60 °C for 72 h until all the water was completely evaporated. The dry weight was measured using an electronic balance and calculated based on the difference between the weight of the sample tube and the empty tube (dry weight = sample tube − empty tube; *n* = 3).

### Protein expression and purification from *E. coli*

*E. coli* BL21 (DE3) cells containing plasmids pET28a-*acs*, pET28a-acs^6K-6Q^, or pET32a-*limB*, respectively, were grown on LB agar with suitable antibiotics. The prepared seed culture was inoculated into LB medium with the corresponding antibiotics by shaking overnight at 37 °C. Next, the seed culture was transferred to TB medium with antibiotics. The culture was induced by adding 0.2 mM IPTG until the optical density at 600 nm (OD_600)_ reached 0.6, followed by induction overnight at 16 °C. The His-tagged LimB, Acs, and Acs^6K-6Q^ proteins were purified using Ni^2+^-NTA resin according to the manufacturer’s instructions (Novagen). The purity of fusion protein was confirmed via SDS-PAGE, and the concentration was measured using the Bradford kit (Sangon Biotech).

### In vitro assays for Acs oxidation catalyzed by LimB and LimB-N

The reaction system consisted of 1 μg of Acs, 2 μg of LimB or LimB-N, 100 μM NADH and 100 μM FAD in a reaction buffer (20 μl) comprising 20 mM Tris-HCI (pH 8.0), 100 mM KCl, 7.5 mM MgCl_2_. The reaction system was incubated at 30 °C for 6 h, followed by analysis of the total loading protein and crotonylation using Western blot with specific antibodies, including anti-Kcr (1:1000) or anti-His antibody (1:2000).

### Immuno-precipitation of 3×FLAG tagged Acs from *S. roseosporus*

The mycelia were harvested and resuspended in PBS through ultrasonication. The clarified lysate was first subjected to centrifugation, then followed by the addition of anti-FLAG M2 affinity gel (Sigma), which was incubated on a roller shaker for 3 h. To remove the supernatant and collect the resin, the mixture was centrifugated at 5000 × *g* for 2 min. The immunoprecipitated complexes were washed three times with PBS before boiling, separated through SDS-PAGE, and analyzed by immunoblotting with either anti-Kcr (1:1000) or anti-FLAG antibody (1:2000).

### In vitro protein degradation assays of Acs

To investigate the degradation of Acs, 10 µg of purified Acs protein was incubated with lysates from WT, *ΔlimB* mutant, and *ΔclpP2* mutant strains, respectively, harvested from 24-h fermentation at 30 °C. The duration of incubation times varied between 0, 0.5, 4, and 8 h. After incubation, samples were denatured through heating, separated through SDS-PAGE, and stained with Coomassie blue.

### Acetyl-CoA synthetase (Acs) activity assays

The activity of Acs was evaluated by incubating 10 µg of purified Acs protein in a reaction (200 µl) containing 100 mM Tris-HCl (pH 7.7), 10 mM MgCl_2_, 10 mM l-malic acid (pH 7.7), 8 mM ATP (pH 7.5), 1 mM NAD, 0.2 mM CoA, 3 U of malic enzyme, and 0.4 U of citrate synthase. The reaction was initiated by adding 100 mM potassium acetate, and the detection signal was performed at 340 nm using a spectrophotometer. Enzyme activity was measured using an extinction coefficient of 6.3 mM^−1^ cm^−1^ for NADH, and one unit of activity was defined as the amount of enzyme required to generate 1 µmol of NADH min^−1^ through the acetate-dependent reaction in the coupling assay^39^.

### Statistics and reproducibility

Statistical analysis of all data in the graphs was performed using Excel 2019 and Prism (version 9.5, GraphPad). All experiments were performed independently, at least in triplicate, and indicated in the corresponding figure legends. Images were combined and annotated in Adobe Photoshop (version 2021).

### Reporting summary

Further information on research design is available in the Nature Portfolio Reporting Summary linked to this article.

### Supplementary information

Supplementary Information

Description of Supplementary Data 1

Supplementary Data 1

Reporting Summary

## Data Availability

All data supporting the findings of the current study within the article and Supplementary Information are available from the corresponding authors upon request. Complete uncropped blots and gels are provided in Supplementary Information Figs. S17–S25. The Source data for the all graphs in Figs. 1c and 6b, c as well as in Supplementary Fig. S2 are shown in Supplementary Data 1.
