# Supplementary material for: Post-crotonylation oxidation by a monooxygenase promotes acetyl-CoA synthetase degradation in Streptomyces roseosporus
Source: Commun Biol. 2023 Dec 8;6:1243. doi: 10.1038/s42003-023-05633-0 (PMC10709465; doi:10.1038/s42003-023-05633-0)
Supplement: Supplementary file 1 — Supplementary Information [file 42003_2023_5633_MOESM1_ESM.pdf]

Supplementary Materials

Post-crotonylation oxidation by a monooxygenase promotes acetyl-CoA synthetase degradation in *Streptomyces roseosporus*

Bing-Bing Ma[1,2#], Chen-Fan Sun[1,2#], Jin-Yi Zhou[1,2#], Shuai-Lei Gu[3], Xin-Yi Dai[3], Yan-Zhen Chen[3], Qing-Wei Zhao[1,4*], Xu-Ming Mao[1,2*]

[1]Department of Clinical Pharmacy, the First Affiliated Hospital & Institute of Pharmaceutical Biotechnology, School of Medicine, Zhejiang University, Hangzhou 310058, China

[2]Zhejiang Provincial Key Laboratory for Microbial Biochemistry and Metabolic Engineering, Hangzhou 310058, China

[3]College of Life Sciences, Zhejiang University, Hangzhou 310058, China

[4]Zhejiang Provincial Key Laboratory for Drug Evaluation and Clinical Research, Hangzhou 310006, China

[#]These authors contributed equally to this work

*These authors jointly supervised this work: qwzhao@zju.edu.cn (Q.-W. Z.), xmmao@zju.edu.cn (X.-M. M.)

Table of Contents

Table S1 Putative luciferase monooxygenases from *S. roseosporus* through Local BLAST using the MsnO8 protein sequence, which corresponds to *S. griseus.* 4

Table S2 The acyl- and acetyl-CoA synthetase libraries and crotonyl lysine modification sites in this study. 5

Table S3 Putative Clp proteases of *S. roseosporus* L30 through Local Blast. 6

Table S4 All strains used in this study..7

Table S5 All plasmids used in this study. 9

Table S6 All primers used in this study. 11

Figure S1 LimB homologs regulates crotonylation..15

Figure S2 Dry weight of wild type (WT), the *limB* null mutant (*ΔlimB*) and *limB* over-expression strain (WT + *ermEp*-limB*) in the YEME culture..16

Figure S3 Protein alignment of LimB from *S. roseosporus* and its homologs Q9X888 from *S. coelicolor* M145, WP_0155508125.1 from *S. albus* J1074 and WP_000130380.1 from *E. coli* DH5α...17

Figure S4 HRMS of daptomycin (a), WT (b), *ΔlimB* (c) and WT + *ermEp*-limB* (d). 18

Figure S5 Proteasome is required for LimB degradation. 19

Figure S6 LimB exerts oxidation on the crotonylation of Acs...20

Figure S7 MS/MS analysis of crotonylation on Acs K116. 21

Figure S8 MS/MS analysis of crotonylation on Acs K133. 22

Figure S9 MS/MS analysis of crotonylation on Acs K367. 23

Figure S10 MS/MS analysis of crotonylation on Acs K496. 24

Figure S11 MS/MS analysis of crotonylation on Acs K593. 25

Figure S12 MS/MS analysis of crotonylation on Acs K605. 26

Figure S13 Bacterial two-hybrid system was used to screen interacting proteins............27

Figure S14 In-frame deletion of *acs* in *S. roseosporus* L30. 28

Figure S15 In-frame deletion of *limB* in *S. roseosporus* L30. 29

Figure S16 In-frame deletion of *clpP2* in *S. roseosporus* L30. 30

Figure S17 Uncropped and unedited blot and gel images for Fig. 1a. 31

Figure S18 Uncropped and unedited blot and gel images for Fig. 3. 32

Figure S19 Uncropped and unedited blot images for Fig. 4b and 4c. 33

Figure S20 Uncropped and unedited blot and gel images for Fig. 5. 34

Figure S21 Uncropped and unedited blot and gel images for Fig. 7b and 7c. 35

Figure S22 Uncropped and unedited blot and gel images for Fig. S1c. 36

Figure S23 Uncropped and unedited blot and gel images for Fig. S5. 37

Figure S24 Uncropped and unedited blot and gel images for Fig. S6b. 38

Figure S25 Uncropped and unedited gel images for Fig. S14, S15 and S16. 39

References 40

Table S1 Putative luciferase monooxygenases from *S. roseosporus* through Local BLAST using the MsnO8 protein sequence, which corresponds to *S. griseus.*

Protein accession	Identities (%)	*S. griseus*
Orf00021	28	WP_012378595.1
Orf00384	32	WP_012378001.1
Orf01706	28	WP_003965970.1
Orf02002	29	WP_012379008.1
Orf03823	26	WP_003967991.1
Orf04334	25	WP_012380442.1
Orf04345	24	WP_003968432.1
Orf04909	39	WP_012380811.1
Orf05584	34	WP_012381147.1
Orf06289	35	WP_012381564.1
Orf06299	34	WP_003970150.1

Table S2 The acyl- and acetyl-CoA synthetase libraries and crotonyl lysine modification sites in this study. Identification and analysis of crotonylated proteins were provided by PTM Biolabs Inc (Hangzhou, China)[1].

Protein accession	Position	Amino acid	Protein description	Modified sequence
Orf01034	69	K	Acyl-CoA synthetase	ALMASGVAK(cr)GDR
Orf01034	433	K	Acyl-CoA synthetase	IK(cr)DMIIR
Orf01034	505	K	Acyl-CoA synthetase	LAHYK(cr)IPR
Orf01035	447	K	Acetyl-CoA synthetase	ADDVFK(cr)ASDYK
Orf03275	116	K	Acetyl-CoA synthetase	AITYAELK(cr)DEVSR
Orf03275	133	K	Acetyl-CoA synthetase	AANALTELGVGK(cr)GDR
Orf03275	367	K	Acetyl-CoA synthetase	TFMK(cr)WGDDIPAK
Orf03275	496	K	Acetyl-CoA synthetase	FEGK(cr)YFAGDGAK
Orf03275	593	K	Acetyl-CoA synthetase	NHVGATLGPIAK(cr)PK
Orf03275	605	K	Acetyl-CoA synthetase	VLPVAELPK(cr)TR
Orf05336	184	K	Acyl-CoA synthetase	GNELLAK(cr)TPDAVTER
Orf05336	330	K	Acyl-CoA synthetase	EYAK(cr)VSQDNFR
Orf05336	353	K	Acyl-CoA synthetase	HK(cr)VADALVFSK
Orf05336	362	K	Acyl-CoA synthetase	VADALVFSK(cr)IR
Orf05795	60	K	Acyl-CoA synthetase	AAAK(cr)GLIASGVQPGDR
Orf05795	194	K	Acyl-CoA synthetase	PK(cr)GCVLTHR
Orf05795	280	K	Acyl-CoA synthetase	VFEK(cr)VYNAAR
Orf05795	294	K	Acyl-CoA synthetase	AQADGK(cr)GK
Orf05795	331	K	Acyl-CoA synthetase	LFDK(cr)LVFGK
Orf05795	552	K	Acyl-CoA synthetase	AVDDGNAAVSK(cr)AESVR

Table S3 Putative Clp proteases of *S. roseosporus* L30 through Local Blast.

Protein accession	Protein description
Orf04819	ATP-dependent Clp protease adapter protein ClpS
Orf04198	ATP-dependent Clp protease
Orf03399	Chaperone protein ClpB
Orf00967	ATP-dependent Clp protease ATP-binding subunit
Orf06758	ATP-dependent Clp protease

Table S4 All strains used in this study.

Strains	Genotype	Reference
S. roseosporus L30	Wild type	This lab[2]
S. coelicolor M145	Wild type	This lab[3]
S. albus J1074	Wild type	This lab[4]
E. coli DH5α	Host for general cloning	Novagen
BTH101	Host for bacterial two-hybrid system	This lab
ET12567/pUZ8002	Conjugation host	This lab
E. coli BL21(DE3)	Host for expression protein	Novagen
ΔprcB/A	In-frame deletion of *prcB/A* in *S. roseosporus*	This lab[1]
ΔclpP2	In-frame deletion of *clpP2* in *S. roseosporus*	This study
ΔlimB	In-frame deletion of *limb* in *S. roseosporus*	This study
Δacs	In-frame deletion of *acs* in *S. roseosporus*	This study
WT + *ermEp*-limB*	*limB* overexpressed under *ermEp** in *S. roseosporus*	This study
WT + *ermEp*-acs*	acs overexpressed under *ermEp** in *S. roseosporus*	This study
WT + *ermEp*-limB (E. coli* DH5α*)*	LimB from *E. coli* DH5α overexpressed under *ermEp** in *S. roseosporus*	This study
WT + *ermEp*-limB (S. coelicolor* M145*)*	*limB* from *S. coelicolor* M145 overexpressed under *ermEp** in *S. roseosporus*	This study
WT + *ermEp*-limB (S. albus* J1074*)*	*limB* from *S. albus* J1074 overexpressed i under *ermEp** in *S. roseosporus*	This study
ΔlimB + *ermEp*-acs*	*acs* overexpressed under *ermEp** in *ΔlimB*	This study
ΔclpP2 + *ermEp*-acs*	*acs* overexpressed under *ermEp** in *ΔclpP2*	This study

ΔprcB/A + *ermEp*-limB*	*limB* overexpressed under *ermEp** in *ΔprcB/A*	This study
LimB	*limB* cloned into pET32a	This study
LimB-N	*limB* cloned into pET32a with bases from 103bp to 591bp	This study
Acs	*acs* cloned into pET28a	This study
$Acs^{6K\text{-}6Q}$	Acs mutated 116K, 133K, 367K, 496K, 593K and 605K to 116Q, 133Q, 367Q, 496Q, 593Q and 605Q, cloned into pET32a	This study

Table S5 All plasmids used in this study.

Plasmids	Genotype	Reference
pKC1139	Bifunctional oriT RK2 plasmid for gene deletion	This lab
pKC1139-*ΔlimB*	*limB* knockout plasmid based on pKC1139	This study
pKC1139-*Δacs*	*acs* knockout plasmid based on pKC1139	This study
pKC1139-*ΔclpP2*	*clpP2* knockout plasmid based on pKC1139	This study
pSN7	Overexpression integrative shuttle vector containing *ermEp** with 3×FLAG tag at N terminus and 18×His tag at C terminus	This lab
pSN7-*limB*	*limB* in pSN7	This study
pSN7-*acs*	*acs* in pSN7	This study
pSN7-*limB* (*E. coli* DH5α)	*limB from E. coli* DH5α in pSN7	This study
pSN7-*limB* (*S. coelicolor* M145)	*limB from S. coelicolor* M145 in pSN7	This study
pSN7-*limB* (*S. albus* J1074)	*limB from S. albus* J1074 in pSN7	This study
pET28a	Protein expression vector containing 6×His label	This lab
pET32a	Protein expression vector containing 6×His label	This lab
pET28a-*acs*	*acs* in pET28a	This study
pET28a-$acs^{6K\text{-}6Q}$	$acs^{6K\text{-}6Q}$ in pET28a	This study
pET32a-*limB*	*limB* in pET32a	This study
pET32a-*limB-N*	*limB-N* in pET32a	This study
pUT18	Vector for bacterial two-hybrid system	This lab
pKT25	Vector for bacterial two-hybrid system	This lab
pUT18-*zip*	Control plasmid for bacterial two-hybrid system	This lab
pKT25-*zip*	Control plasmid for bacterial two-hybrid system	This lab
pUT18-*limB*	*limB in pUT18*	This study
pUT18-*limB-N*	*limB-N* in pUT18	This study

pKT25-*orf1034*	*orf1034* in pKT25	This study
pKT25-*orf1035*	*orf1035* in pKT25	This study
pKT25-*orf3275*	*orf3275* in pKT25	This study
pKT25-*orf5336*	*orf5336* in pKT25	This study
pKT25-*orf5795*	*orf5795* in pKT25	This study
pUT18-*clpP1*	*clpP1* in pUT18	This study
pUT18-*clpP2*	*clpP2* in pUT18	This study
pUT18-*prcA*	*prcA* in pUT18	This study
pUT18-*prcB*	*prcB* in pUT18	This study
pUT18-*ftsH*	*ftsH* in pUT18	This study
pUT18-*lon*	*lon* in pUT18	This study
pUT18-*pup*	*pup* in pUT18	This study
pUT18-*clpA*	*clpA* in pUT18	This study
pUT18-*clpB*	*clpB* in pUT18	This study
pUT18-*clpX*	*clpX* in pUT18	This study
pUT18-*orf967*	*orf967* in pUT18	This study
pUT18-*orf3399*	*orf3309* in pUT18	This study
pUT18-*orf4198*	*orf4198* in pUT18	This study
pUT18-*orf4819*	*orf4819*in pUT18	This study
pUT18-*orf6758*	*orf6758* in pUT18	This study

Table S6 All primers used in this study.

Plasmids	No.	Sequence
pKC1139-*ΔlimB*	1	CAGGTCGACTCTAGAGGATCCGAAGAAGTTCACGGTGAAG
	2	GTGGAGTTCATGCGATTCTGTGATCGAACAGTGTTCAC
	3	GTGAACACTGTTCGATCACAGAATCGCATGAACTCCAC
	4	CTATGACATGATTACGAATTCCTCGCCGACTTCCTGGACG
pKC1139-*Δacs*	5	GGCTGCAGGTCGACTCTAGAGCTACTCGGCGGGCTTCT
	6	AGGAGTCGGTCAGCGTCGTGCAGGGTGTCCGTTGTGTCC
	7	GGACACAACGGACACCCTGCACGACGCTGACCGACTCCT
	8	GATATCGCGCGCGGCCGCCCGTCTTCGACACATCCGATGTC
pKC1139-*ΔclpP2*	9	CAGGTCGACTCTAGAGGATCCACTACTCCGGCATCGAGGTC
	10	GCCAAGGGGAAAGACCGACGGTGGGTGTTCACCATCCTGG
	11	CCAGGATGGTGAACACCCACCGTCGGTCTTTCCCCTTGGC
	12	CTATGACATGATTACGAATTCGCCGGTGCTCAGCGCACCGC
pSN7-*limB*	13	GCGGCGGGGGCGGCAGATCTGTGGAGTTCATGCGATTCTC
	14	GTCTAGAAGATCGATGTGATTCACTCCCGGTCGATCACTC
pSN7-*acs*	15	GGATATCACATCGATCTTCTAGAGTGAGCAACGAGAGC

		CT
	16	CCCCCGCCGCCGCCGGATCCTCAGTCCTCGTTGGTCGAGG
pSN7-*limB (E. coli* DH5α*)*	17	GCGGCGGGGGCGGCAGATCTATGACTGATAAAACCATTGC
	18	GGTCTAGAAGATCGATGTGATCTATCCCAACAACTCTTCC
pSN7-*limB (S. coelicolor* M145*)*	19	GGCGGGGGCGGCAGATCTATGCAGTTCGGCATCTTCACCG
	20	GGTCTAGAAGATCGATGTGATTCATGCGCGCGCTCCTTCC
pSN7-*limB (S. albus* J1074*)*	21	GCGGCGGGGGCGGCAGATCTATGAGATTCCAGGTGCTCTCC
	22	GTCTAGAAGATCGATGTGATTCAGCCCCAGAGCGTCGTCGGC
pET28a-*acs*	23	GGTGCCGCGCGGCAGCCATATGGTGAGCAACGAGAGCC
	24	CGAGTGCGGCCGCAAGCTTTCAGTCCTCGTTGGTCG
pET28a-$acs^{6K\text{-}6Q}$	25	CACCTACGCGGAGCTGTGAGACGAGGTCTCCCGGGC
	26	GCCCGGGAGACCTCGTCTCACAGCTCCGCGTAGGTG
	27	AGCTGGGTGTCGGCAAGGGCGACCGGGTCGCCGTCTATC
	28	GATAGACGGCGACCCGGTCGCCCTTGCCGACACCCAGCT
	29	CGATCCGTACGTTCATGTGATGGGGCGACGACATCC
	30	GGATGTCGTCGCCCCATCACATGAACGTACGGATCG
pET32a-*limB*	31	TGTCGACGGAGCTCGAATTCTCACTCCCGGTCGATCACTCC
	32	CCATGGCTGATATCGGATCCGTGGAGTTCATGCGATTC

		TCC
pET32a-*limB-N*	33	CTGATATCGGATCCATGGATACGGCCTCGGTGGC
	34	TCGAGTGCGGCCGCAAGCTTTCAGTCGCCGTGTTTCG CC
pUT18-*limB*	35	CATGATTACGCCAAGCTTGGTGGAGTTCATGCGATT
	36	GGCTGAATTCGAGCTCGGTACCTCACTCCCGGTCGA
pUT18-*limB-N*	37	TTACGCCAAGCTTGGTGGAGTTCATGCGATTCT
	38	GAATTCGAGCTCGGTACCTCATTGTCGATGATCTGC
pKT25-*orf1034*	39	TGCAGGGTCGACTCTAGAGGTGAGCCCGCTCTCGTA
	40	TAGTTACTTAGGTACCCGTCAGGAGTCCTGGGTGTCCC
pKT25-*orf1035*	41	GCTGCAGGGTCGACTCTAGAGATGTCGGCAACCAGC
	42	AGTTACTTAGGTACCCGTCACTTCAGGTCTCCCTCG
pKT25-*orf3275*	43	TGCAGGGTCGACTCTAGAGGTGAGCAACGAGAGCCTG
	44	TCTTAGTTACTTAGGTACCCGTCAGTCCTCGTTGGTCG
pKT25-*orf5336*	45	CAGGGTCGACTCTAGAGGTGAGCGACACACAGACCTT
	46	TTAGTTACTTAGGTACCCGTCAGGCCTCGCGCGAGC
pKT25-*orf5795*	47	CAGGGTCGACTCTAGAGTTGCGCGAGTTCAGTCTTCC
	48	AGTTACTTAGGTACCCGTCAGGCGCGGTAGATCGAC
pUT18-*clpP1*	49	TTACGCCAAGCTTGGTGACGAATCTGATGCCCTACGC
	50	TGAATTCGAGCTCGGTACCGGCACCGGTGCCGCC
pUT18-*clpP2*	51	ACGCCAAGCTTGATGAACAACTTCTCCGGCG
	52	AATTCGAGCTCGGTACCGAGCGATGCGCCC
pUT18-*prcA*	53	GTGTCGACGCCGTTCTATGT
	54	CTACTTGTCGGTCTCCTCCGAGGA
pUT18-*prcB*	55	GTGGAAGCCAACACTCGTAG
	56	GAGCAGCGCGGCGCGCGG
pUT18-*ftsH*	57	ATGACTAACGAGTCCCAGGC
	58	GGCCGCGGCCGGGATCCTTT
pUT18-*lon*	59	ATGTGGATCGTGCTGGC

	60	CTAGCTCTCGGGACGGT
pUT18-*pup*	61	ATGGCGACCAAGGACACC
	62	CTACTCCCCGCCCTTTTG
pUT18-*clpA*	63	TTACGCCAAGCTTGATGTCGATGGCGT
	64	TTCGAGCTCGGTACCCTCCTCGCCGTC
pUT18-*clpB*	65	ACCATGATTACGCCAAGCTTGGTGGACGCCGAGCT
	66	TGAATTCGAGCTCGGTACCCGAGGCGGGGCCGAC
pUT18-*clpX*	67	TACGCCAAGCTTGGTGGCACGCATC
	68	AGCTCGGTACCCGCGGATTTCTCGTG
pUT18-*orf967*	69	ATGATTACGCCAAGCTTGATGACGCAGCCTC
	70	TGAATTCGAGCTCGGTACCGGGCTTCTGC
pUT18-*orf3399*	71	ACCATGATTACGCCAAGCTTGGTGGACGCCGAGCT
	72	TGAATTCGAGCTCGGTACCCGAGGCGGGGCCGAC
pUT18-*orf4198*	73	AAGCTTGATGTTCGAGAGGTTCACCGAC
	74	AATTCGAGCTCGGTACCCGCGTCCTTCGTCA
pUT18-*orf4819*	75	TTACGCCAAGCTTGATGGGACAGGTGAG
	76	TTCGAGCTCGGTACCCTAGTTGCGGTCC
pUT18-*orf6758*	77	TTACGCCAAGCTTGATGATCCGCCCCGCC
	78	CGAGCTCGGTACCGCGGTTCTCCACGACGTG

Figure S1 LimB homologs regulates crotonylation.

a. The epoxidation reaction catalyzed by MsnO8 for the biosynthesis of mensacarcin.

b. Domain organization of MsnO8 and LimB. The Flavin_utilizing_monooxygenase superfamily domain was shown in both proteins.

c. *S. roseosporus* LimB (Orf6299) and its homologs from *S. coelicolor* M145 (Q9X888), *S. albus* J1074 (WP_0155508125.1) and *E. coli* DH5α (WP_000130380.1) were expressed in *S. roseosporus* L30 under the promoter *ermEp** and tagged with 3×FLAG. The cell lysate of WT and recombinant strains was prepared and subject for Western blot with α-Kcr or α-FLAG antibody, and the total protein was stained with Coomassie blue for the loading control.

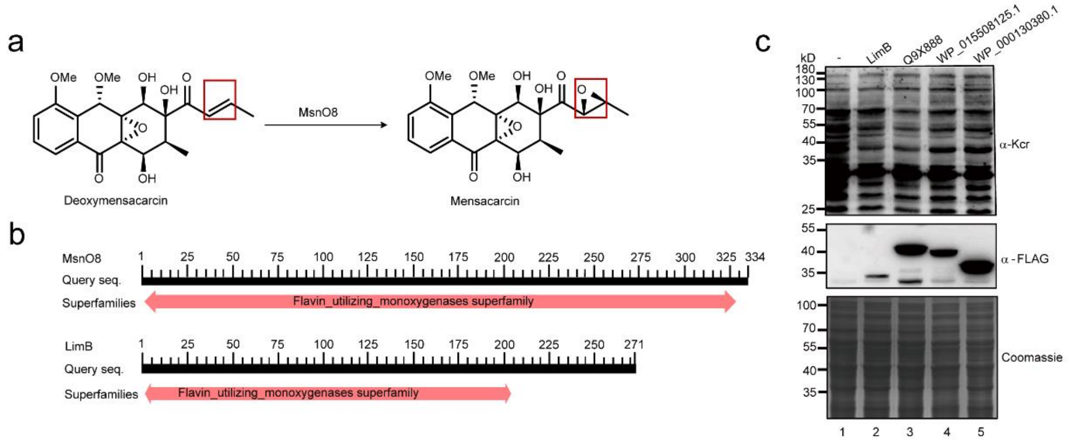

Figure S2 Dry weight of wild type (WT), the *limB* null mutant (*ΔlimB*) and *limB* over-expression strain (WT + *ermEp*-limB*) in the YEME culture. Data are mean ± SEM for n = 3 biologically independent samples.

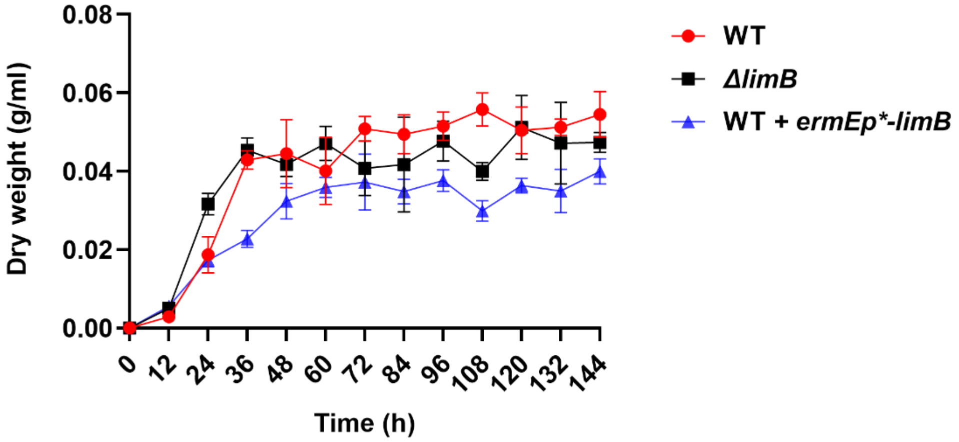

Figure S3 Protein alignment of LimB from *S. roseosporus* and its homologs Q9X888 from *S. coelicolor* M145, WP_0155508125.1 from *S. albus* J1074 and WP_000130380.1 from *E. coli* DH5α.

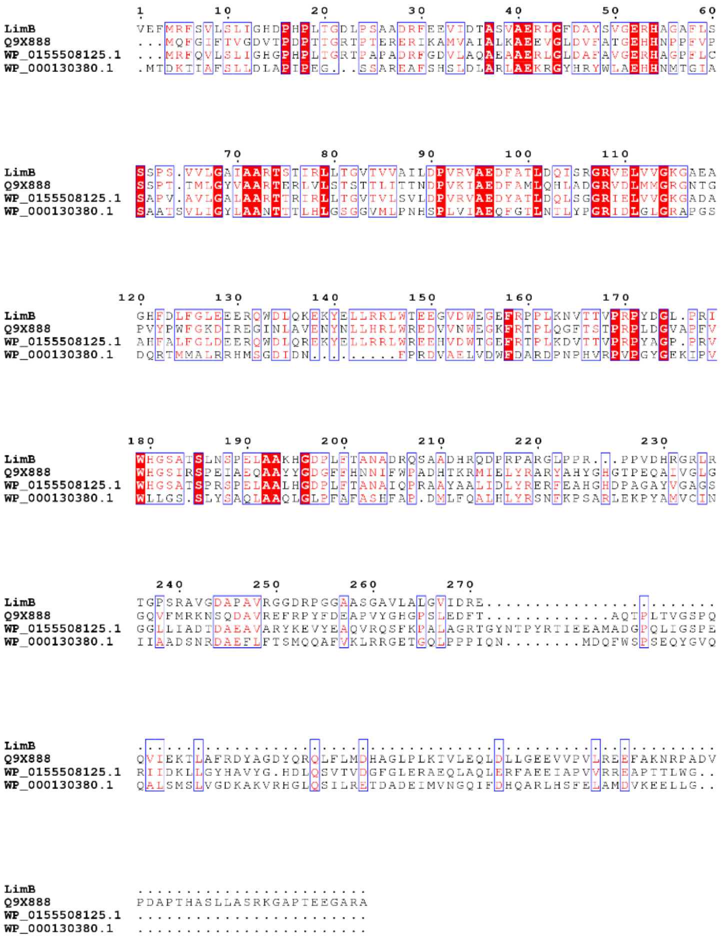

Figure S4 HRMS of daptomycin (a), WT (b), *ΔlimB* (c) and WT + *ermEp*-limB* (d).

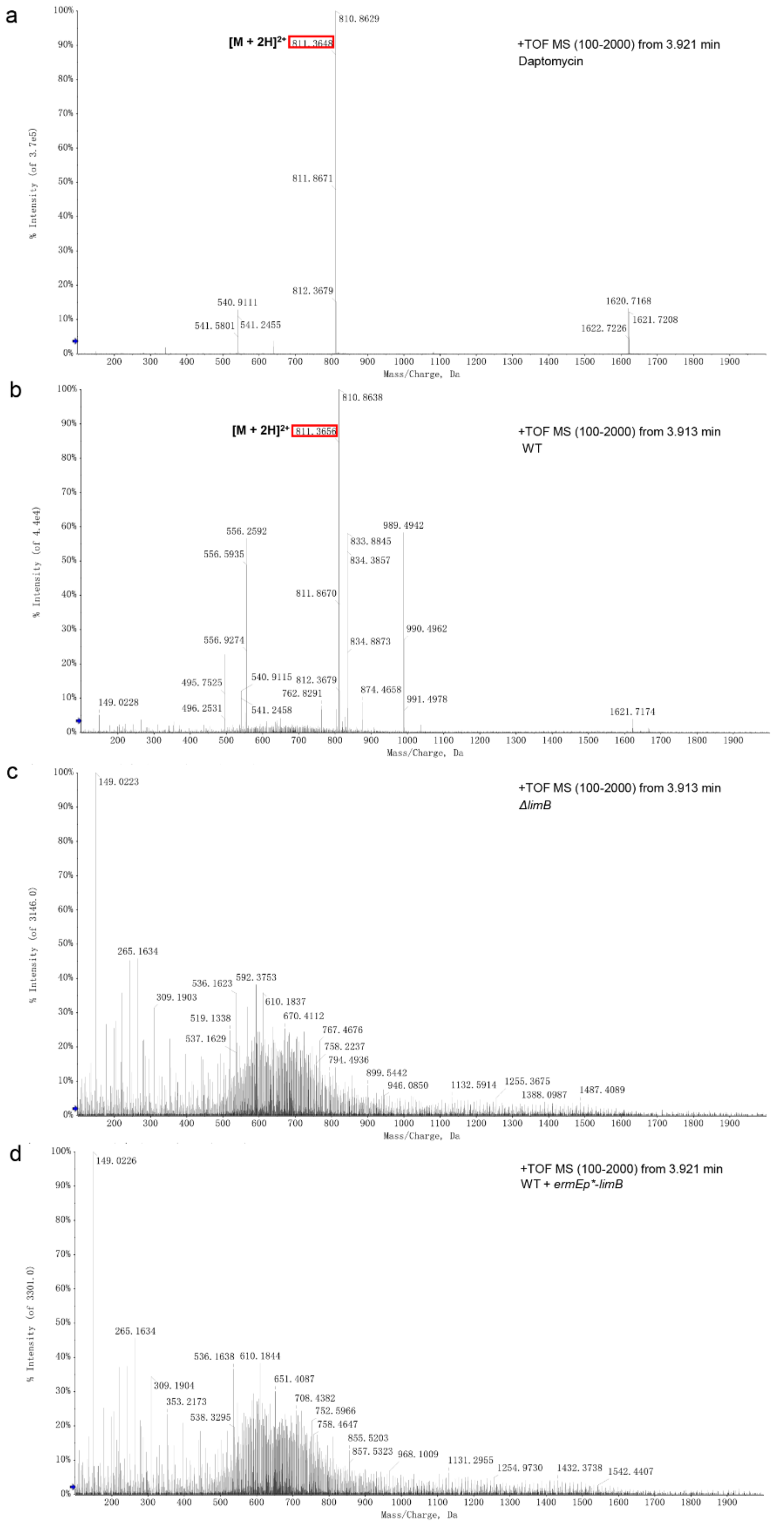

Figure S5 Proteasome is required for LimB degradation.

3×FLAG-tagged LimB was expressed in WT and the *ΔprcB/A* mutant, respectively, and the total cell lysate was prepared and subject to Western blot with an α-FLAG antibody or Coomassie blue staining as the loading control.

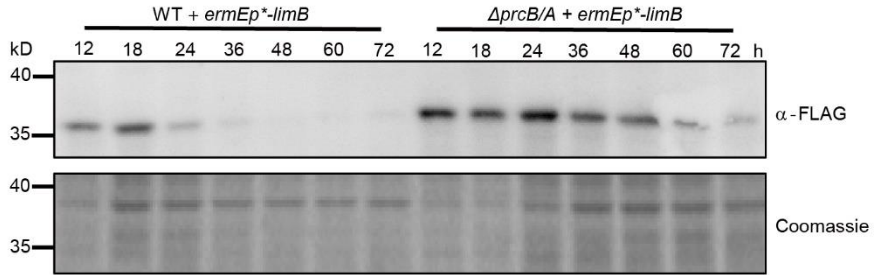

Figure S6 LimB exerts oxidation on the crotonylation of Acs.

a. Amino acid sequence of Acs was shown. Six identified Lys residues for crotonylation were bold and underlined.

b. *In vitro* oxidation assays with purified His-tagged LimB and Acs mutant (Acs^{6K-6Q}). Acs^{6K-6Q} was incubated LimB and Western blot assays were demonstrated with α-Kcr antibody for the crotonylation level and α-His antibody for the loading control of Acs^{6K-6Q}. The control assays without LimB or coenzymes (NADH, FAD) or neither of them were also included (lane 1-3).

a

MSNESLANLLREERKFAPPAELAANANVTAEAYEQAEADRLGFWAEQARRLTWATEPTETLD
WSNPPFAKWFADGKLNVAYNCVDRHVEAGNGDRVAIHFEGEPGDSRAITYAEL**K**DEVSRAAN
ALTELGVG**K**GDRVAVYLPMIPEAAVAMLACARIGAAHSVVFGGFSADAIAARIKDADAKVVI
TADGGYRRGKPSALKPAVDDAVSRFDTVEHVLVVRRTGQDTAWTEGRDIWWHEITARQSAEH
TPEAFDAEQPLFILYTSGTTGKPKGILHTSGGYLTQASYTHHAVFDLKPESDVYWCTADIGW
VTGHSYIVYGPLANGATQVMYEGTPDTPHQGRFWEIVQKYGVTILYTAPTAIRTFM**K**WGDDI
PAKFDLSSLRVLGSVGEPINPEAWMWYRKNIGADKCPIVDTWWQTETGAMMISPLPGVTETK
PGSAQRALPGISATVVDDEANEVPNGGGGYLVLTEPWPSMLRTIWGDDQRFIDTYWSRFEG**K**
YFAGDGAKKDEDGDVWLLGRVDDVMLVSGHNISTTEVESALVSHPSVAEAAVVGAADETTGQ
AIVAFVILRGTATASDELVADLRNHVGATLGPIA**K**PKRVLPVAELP**K**TRSGKIMRRLLRDVA
ENRELGDVTTLTDSSVMDLITTQLPSSSTNED

b

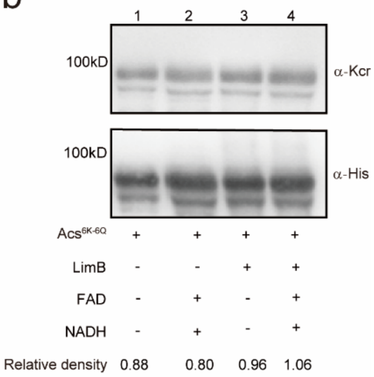

Figure S7 MS/MS analysis of crotonylation on Acs K116. Crotonylation was calculated based on the molecular weight difference values between y6 and y5 (801.41 - 605.29 = 196.12).

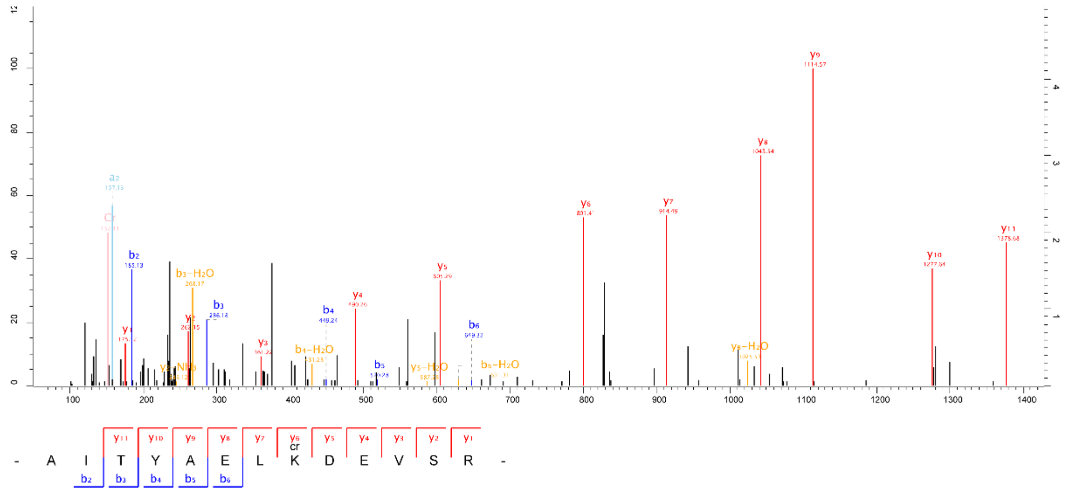

Figure S8 MS/MS analysis of crotonylation on Acs K133. Crotonylation was calculated based on the molecular weight difference values between y4 and y3 (543.29 - 347.17 = 196.12).

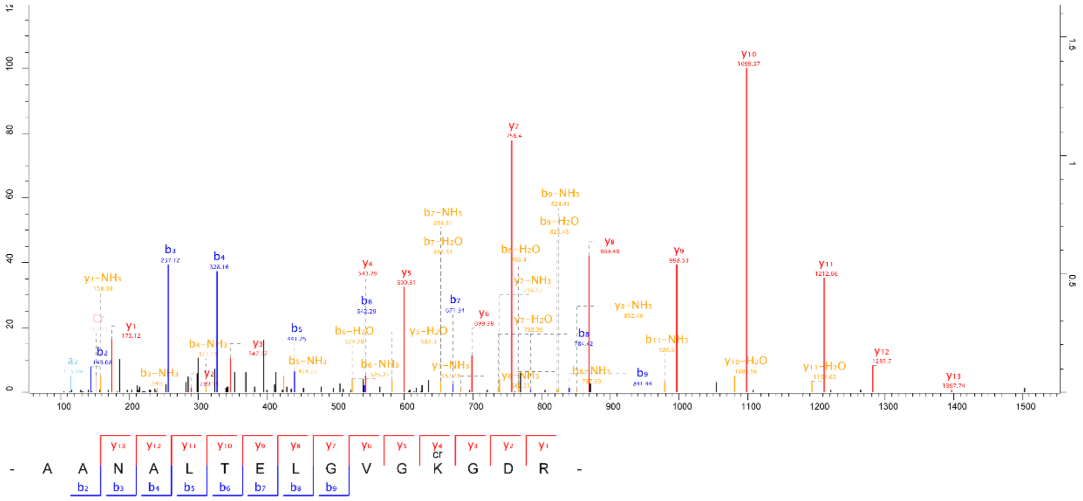

Figure S9 MS/MS analysis of crotonylation on Acs K367. Crotonylation was calculated based on the molecular weight difference values between y9 and y8 (1097.56 - 901.44 = 196.12).

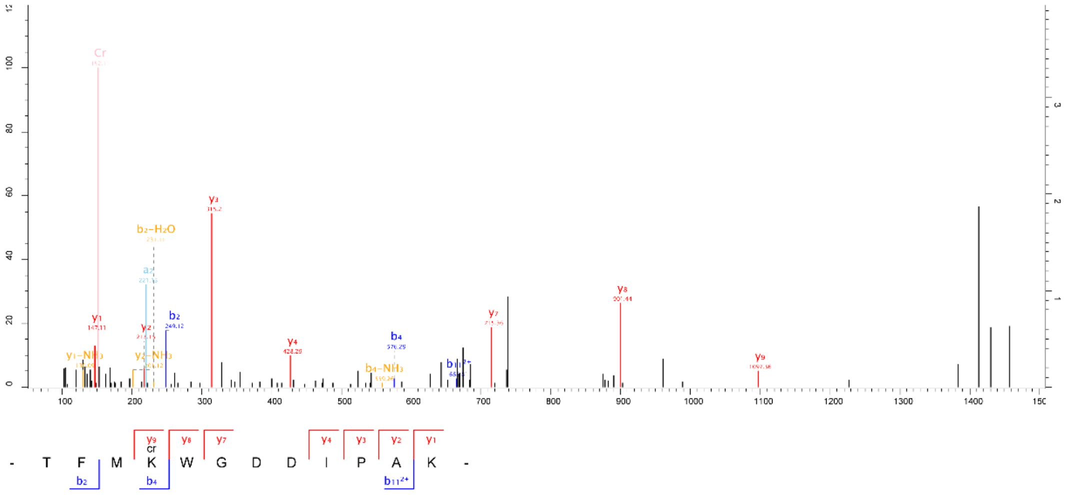

Figure S10 MS/MS analysis of crotonylation on Acs K496. Crotonylation was calculated based on the molecular weight difference values between y9 and y8 (1024.51 - 828.39 = 196.12).

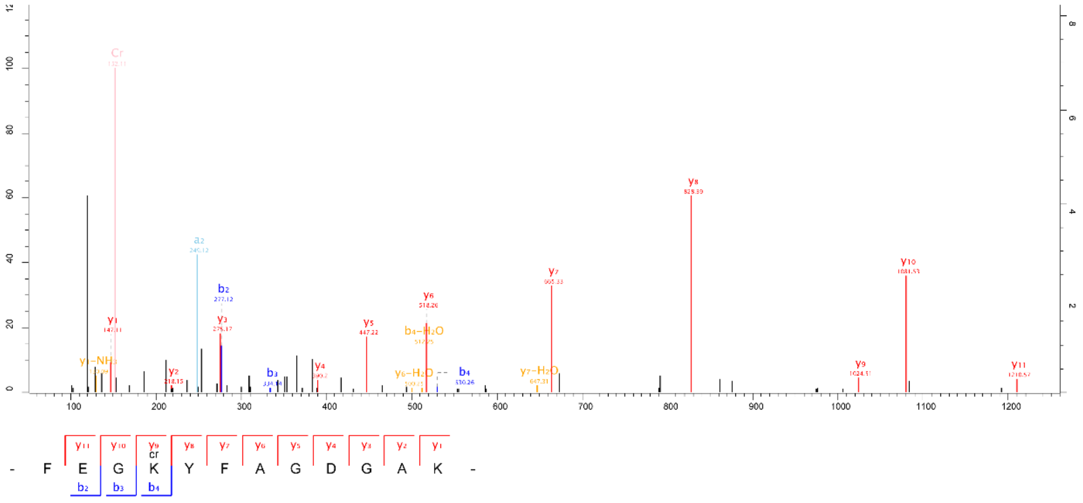

Figure S11 MS/MS analysis of crotonylation on Acs K593. Crotonylation was calculated based on the molecular weight difference values between y3 and y2 (440.29 - 244.17 = 196.12).

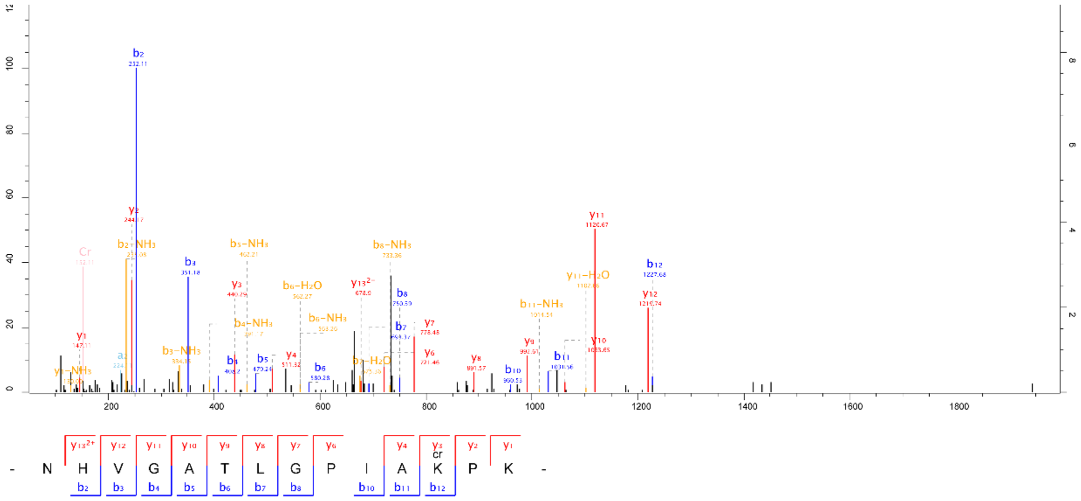

Figure S12 MS/MS analysis of crotonylation on Acs K605. Crotonylation was calculated based on the molecular weight difference values between y6, y5 and b3, b2 ((569.34 - 276.17) - (310.21 - 213.16) = 196.12).

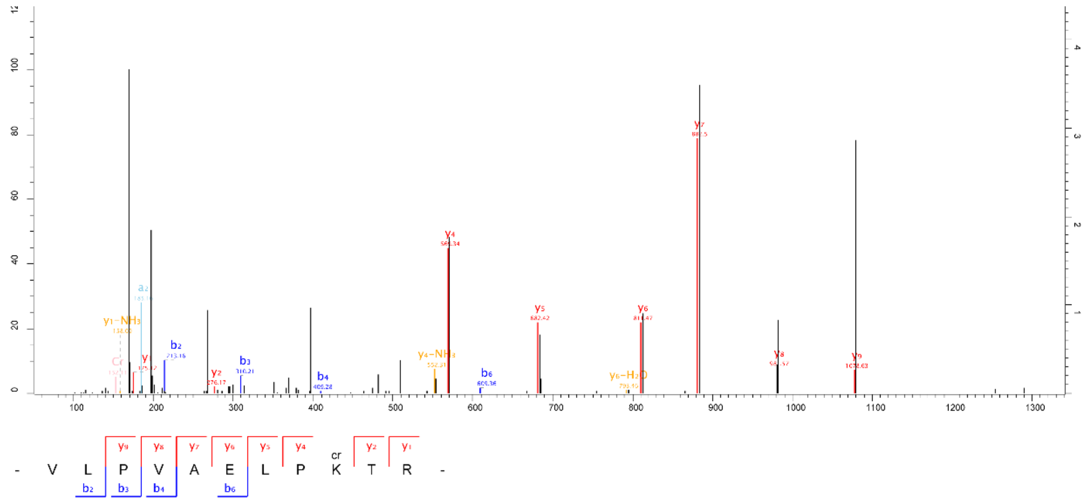

Figure S13 Bacterial two-hybrid system was used to screen interacting proteins. The *acs* gene was cloned into pKT25, and *clpP1, clpP2, clp A, clpB, lon, ftsH, clpX, prcA, prcB, pup, orf967, orf3399, orf4198, orf4819 and orf6758* were cloned into pUT18. The empty plasmids were used as a negative control. The *zip* plasmids were used as a positive control.

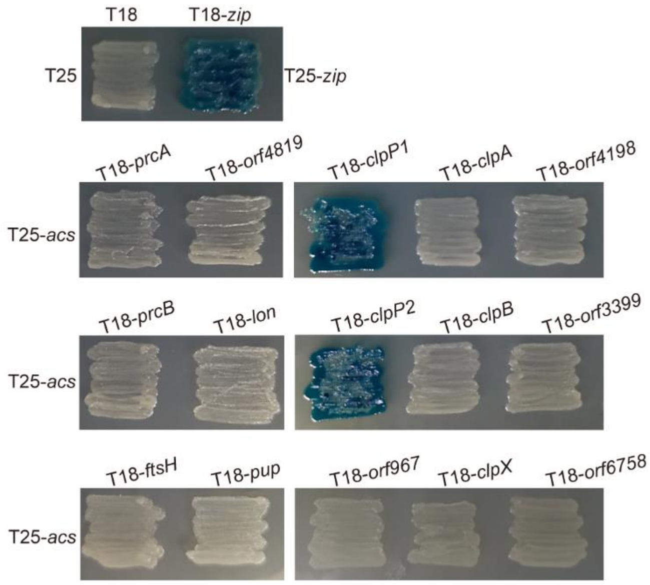

Figure S14 In-frame deletion of *acs* in *S. roseosporus* L30.

a. Schematic diagram of *acs* knock-out. The expected DNA fragment sizes were shown.

b. Confirmative PCR for *acs* deletion. The fragments of 3.2 kb and 1.2 kb were amplified from the genomic DNA of wild type and the *Δacs* mutant, respectively.

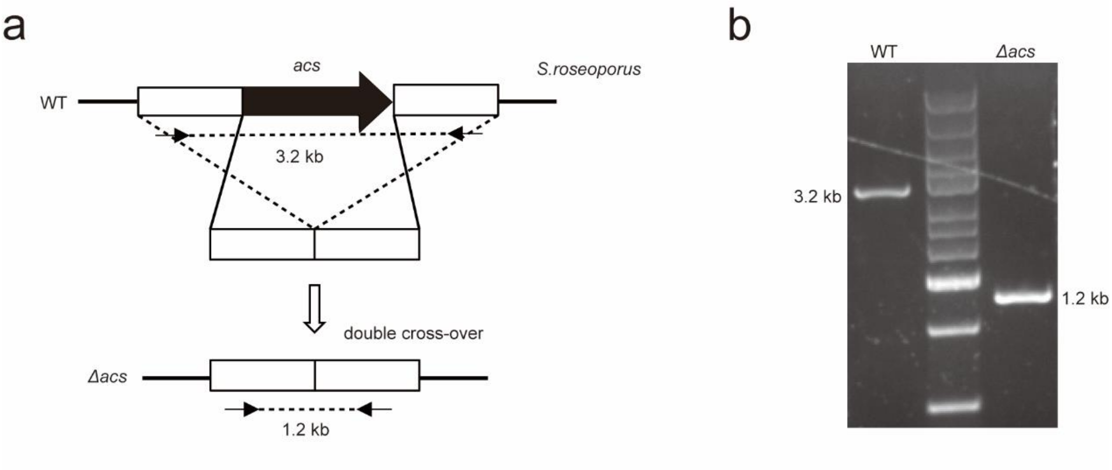

Figure S15 In-frame deletion of *limB* in *S. roseosporus* L30.

a. Schematic diagram of *limB* knock-out. The expected DNA fragment sizes were shown.

b. Confirmative PCR for *limB* deletion. The fragments of 1.8 kb and 1.0 kb were amplified from the genomic DNA of wild type and the *ΔlimB* mutant, respectively.

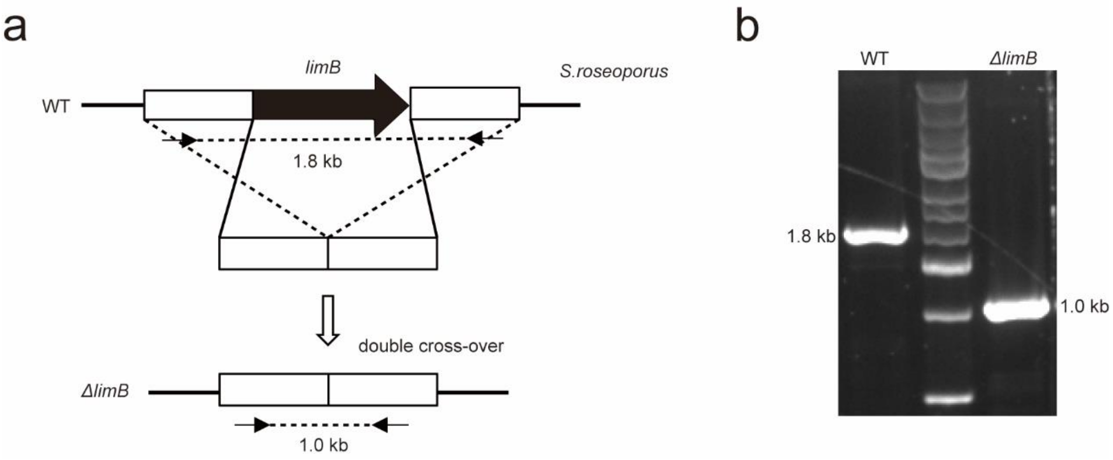

Figure S16 In-frame deletion of *clpP2* in *S. roseosporus* L30.

a. Schematic diagram of *clpP2* knock-out. The expected DNA fragment sizes were shown.

b. Confirmative PCR for *clpP2* deletion. The fragments of 1.6 kb and 1.0 kb were amplified from the genomic DNA of wild type and the *ΔclpP2* mutant, respectively.

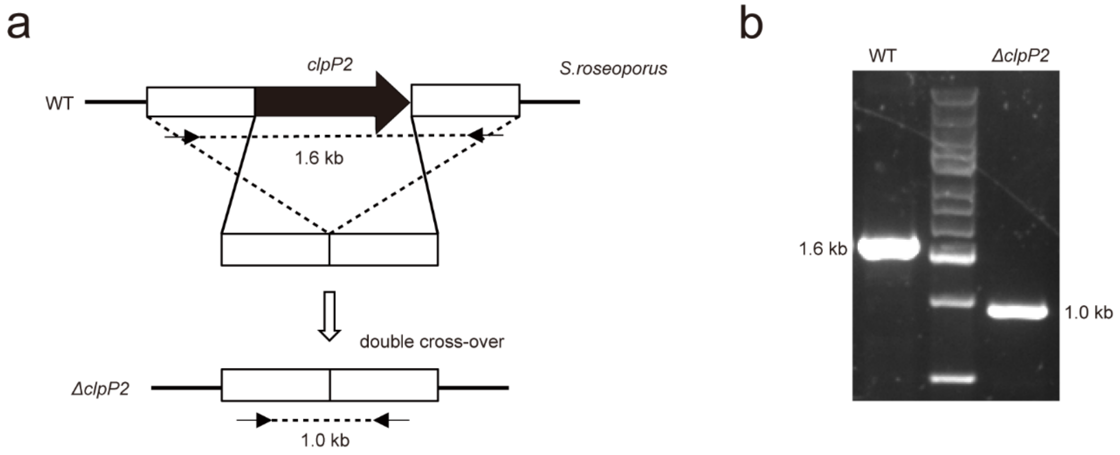

Figure S17 Uncropped and unedited blot and gel images for Fig. 1a.

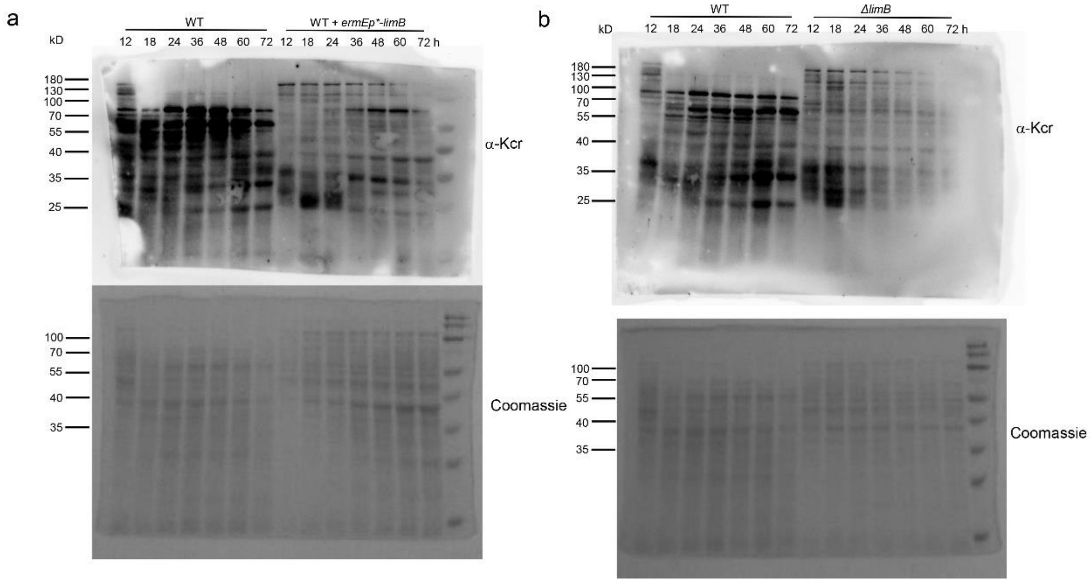

Figure S18 Uncropped and unedited blot and gel images for Fig. 3.

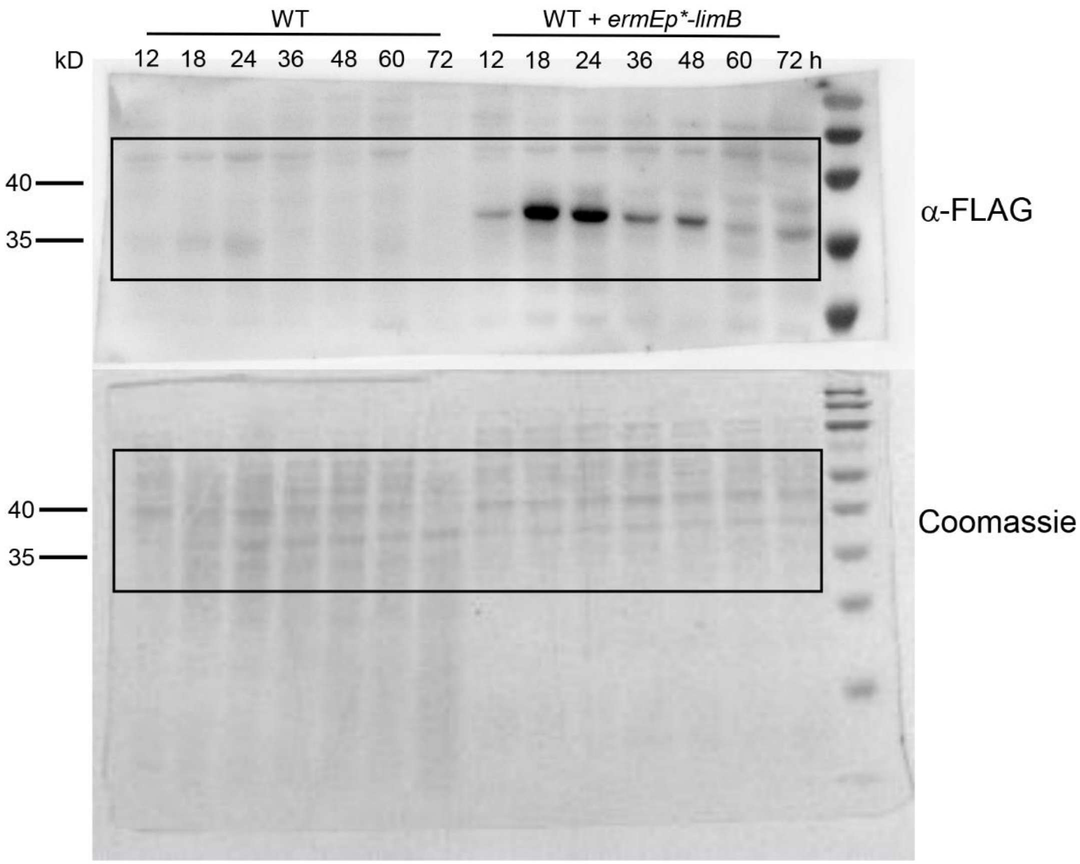

Figure S19 Uncropped and unedited blot images for Fig. 4b and 4c.

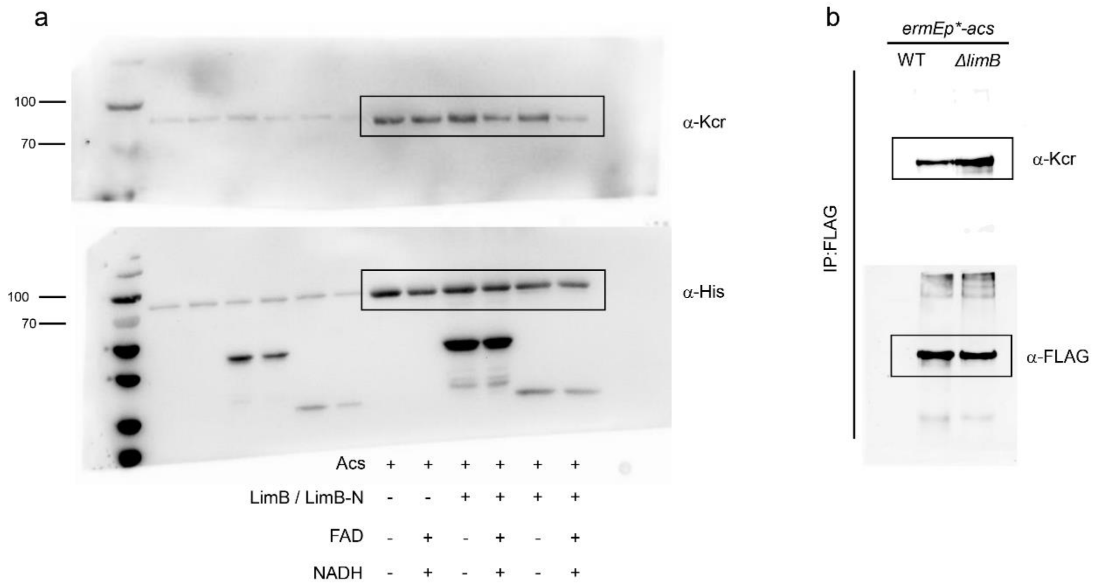

Figure S20 Uncropped and unedited blot and gel images for Fig. 5.

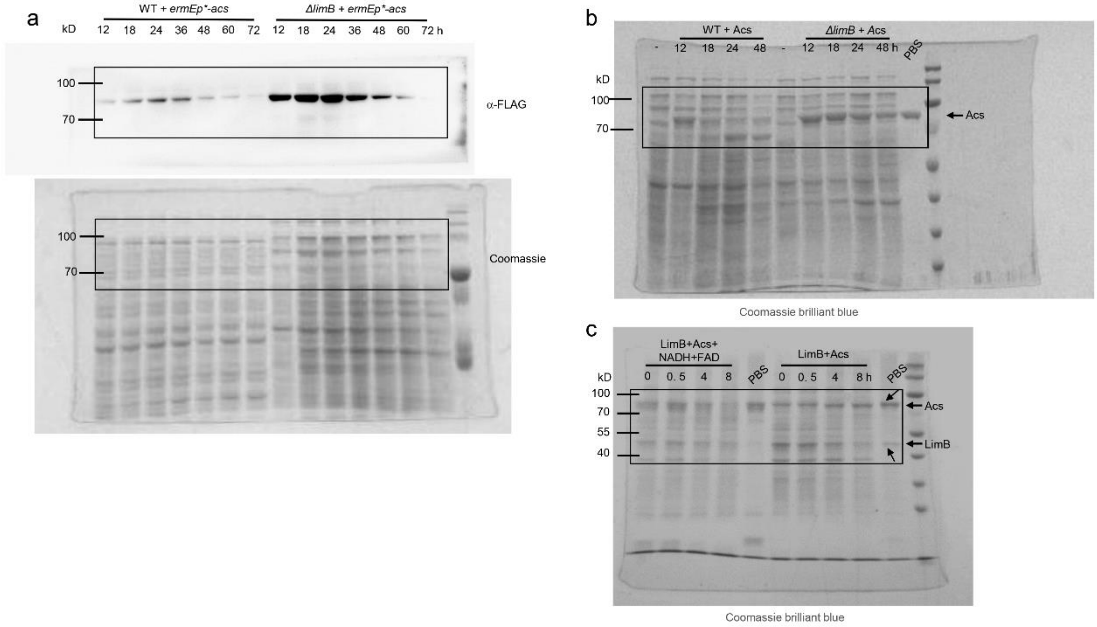

Figure S21 Uncropped and unedited blot and gel images for Fig. 7b and 7c.

a

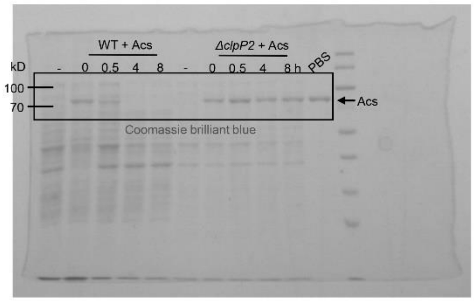

b

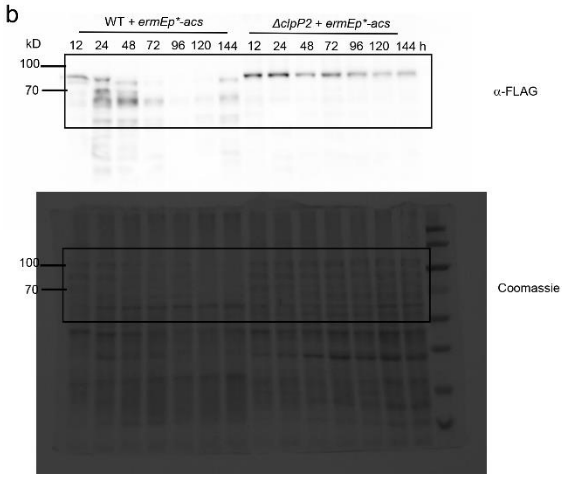

Figure S22 Uncropped and unedited blot and gel images for Fig. S1c.

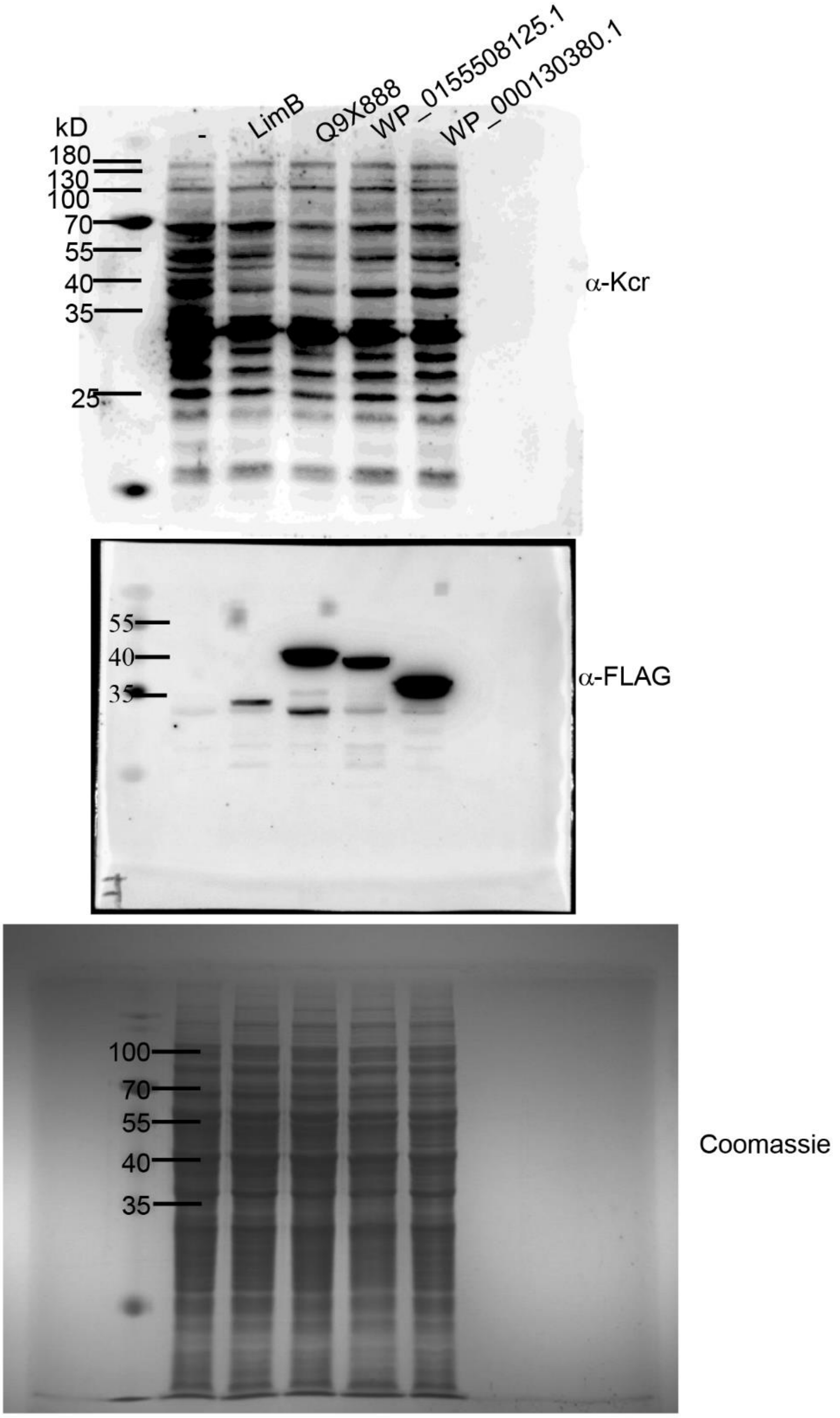

Figure S23 Uncropped and unedited blot and gel images for Fig. S5.

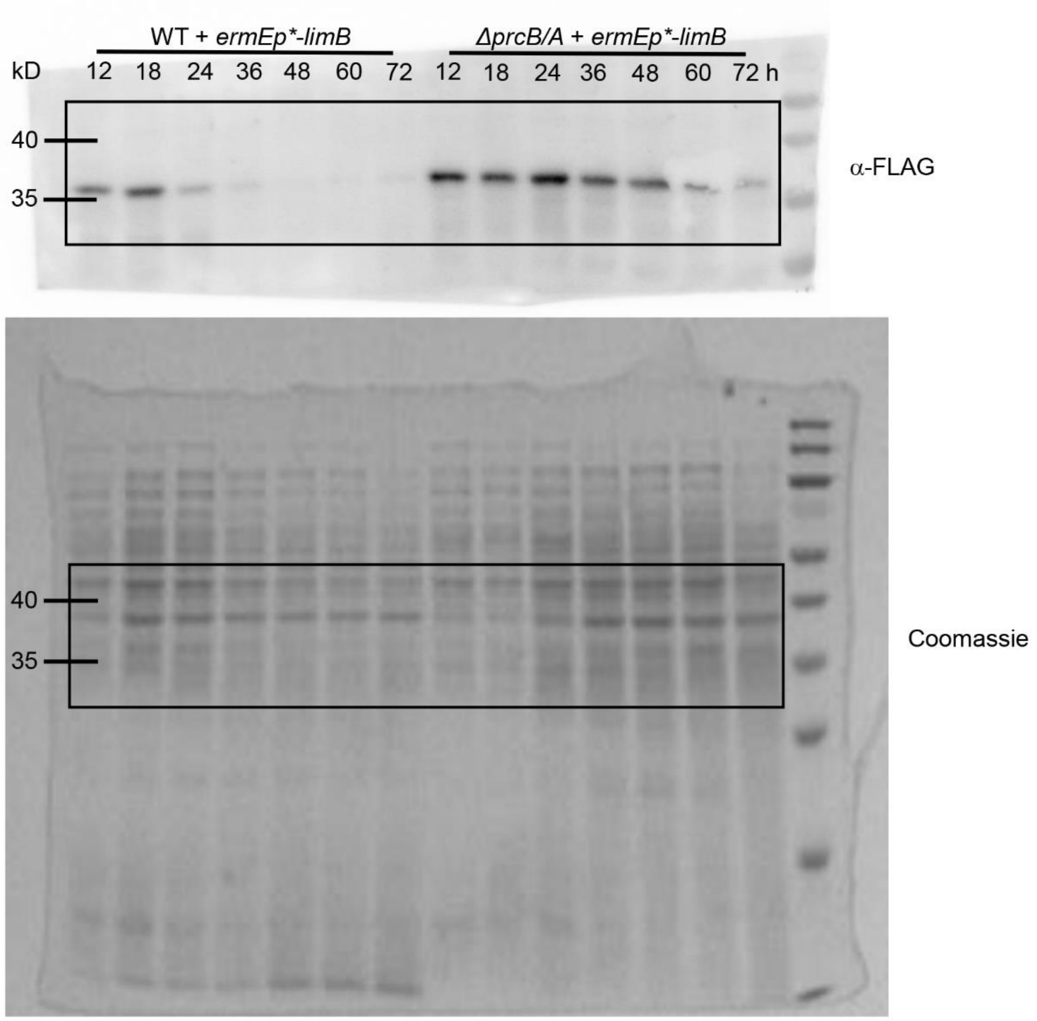

Figure S24 Uncropped and unedited blot and gel images for Fig. S6b.

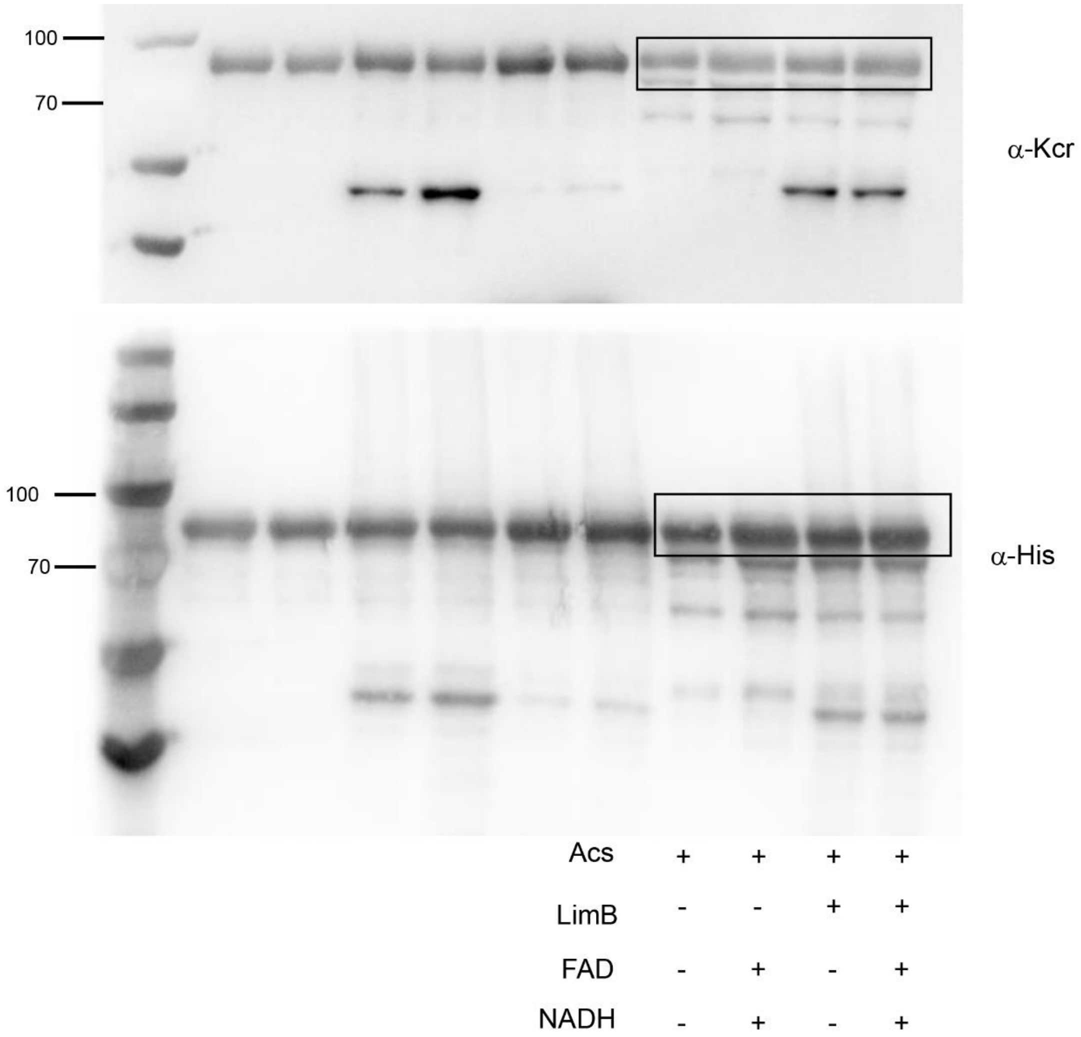

Figure S25 Uncropped and unedited gel images for Fig. S14, S15 and S16.

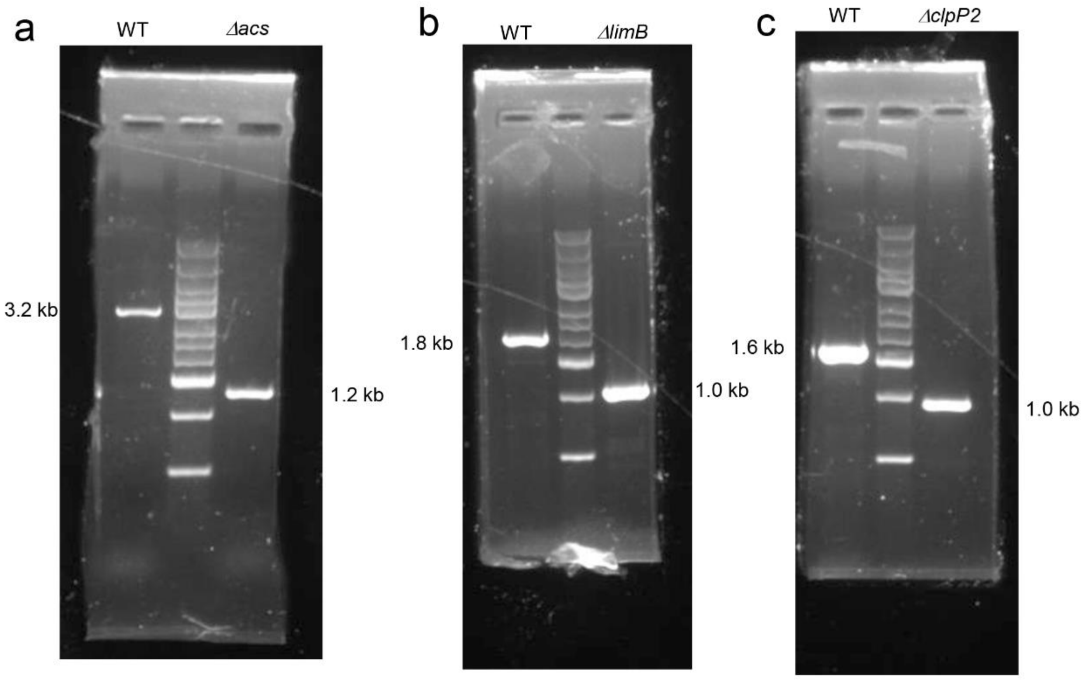

References

1 Sun, C. F., Xu, W. F., Zhao, Q. W., Luo, S., Chen, X. A., Li, Y. Q. & Mao, X. M. Crotonylation of key metabolic enzymes regulates carbon catabolite repression in *Streptomyces roseosporus*. *Commun Biol* **3**, 192, doi:10.1038/s42003-020-0924-2 (2020).

2 Mao, X. M., Luo, S., Zhou, R. C., Wang, F., Yu, P., Sun, N., Chen, X. X., Tang, Y. & Li, Y. Q. Transcriptional regulation of the daptomycin gene cluster in *Streptomyces roseosporus* by an autoregulator, AtrA. *J Biol Chem* **290**, 7992-8001, doi:10.1074/jbc.M114.608273 (2015).

3 Mao, X. M., Ren, N. N., Sun, N., Wang, F., Zhou, R. C., Tang, Y. & Li, Y.Q. Proteasome involvement in a complex cascade mediating SigT degradation during differentiation of *Streptomyces coelicolor*. *FEBS Lett* **588**, 608-613, doi:10.1016/j.febslet.2013.12.029 (2014).

4 Zaburannyi, N., Rabyk, M., Ostash, B., Fedorenko, V. & Luzhetskyy, A. Insights into naturally minimised *Streptomyces albus* J1074 genome. *BMC Genomics* **15**, 97, doi:10.1186/1471-2164-15-97 (2014).
