# Supplementary material for: Post-crotonylation oxidation by a monooxygenase promotes acetyl-CoA synthetase degradation in Streptomyces roseosporus
Source: Commun Biol. 2023 Dec 8;6:1243. doi: 10.1038/s42003-023-05633-0 (PMC10709465; doi:10.1038/s42003-023-05633-0)
Supplement: Supplementary file 2 — Description of Supplementary Data 1 [file 42003_2023_5633_MOESM2_ESM.docx]

**Description of Additional Supplementary Files**

**File name:** Supplementary Data 1

**Description:** the raw/source data behind graphs in the main Figure 1c, 6b and 6c, as well as the Supplementary Figure S2.
