# Supplementary material for: Post-crotonylation oxidation by a monooxygenase promotes acetyl-CoA synthetase degradation in Streptomyces roseosporus
Source: Commun Biol. 2023 Dec 8;6:1243. doi: 10.1038/s42003-023-05633-0 (PMC10709465; doi:10.1038/s42003-023-05633-0)
Supplement: Supplementary file 4 — Reporting Summary [file 42003_2023_5633_MOESM4_ESM.pdf]

nature portfolio

Corresponding author(s): Xu Ming Mao, Qing Wei Zhao

Last updated by author(s): Nov 3, 2023

Reporting Summary

Nature Portfolio wishes to improve the reproducibility of the work that we publish. This form provides structure for consistency and transparency in reporting. For further information on Nature Portfolio policies, see our Editorial Policies and the Editorial Policy Checklist.

Statistics

For all statistical analyses, confirm that the following items are present in the figure legend, table legend, main text, or Methods section.

n/a	Confirmed	
☐	☒	The exact sample size (*n*) for each experimental group/condition, given as a discrete number and unit of measurement
☐	☒	A statement on whether measurements were taken from distinct samples or whether the same sample was measured repeatedly
☐	☒	The statistical test(s) used AND whether they are one- or two-sided<br>*Only common tests should be described solely by name; describe more complex techniques in the Methods section.*
☒	☐	A description of all covariates tested
☒	☐	A description of any assumptions or corrections, such as tests of normality and adjustment for multiple comparisons
☐	☒	A full description of the statistical parameters including central tendency (e.g. means) or other basic estimates (e.g. regression coefficient) AND variation (e.g. standard deviation) or associated estimates of uncertainty (e.g. confidence intervals)
☐	☒	For null hypothesis testing, the test statistic (e.g. *F*, *t*, *r*) with confidence intervals, effect sizes, degrees of freedom and *P* value noted<br>*Give P values as exact values whenever suitable.*
☒	☐	For Bayesian analysis, information on the choice of priors and Markov chain Monte Carlo settings
☒	☐	For hierarchical and complex designs, identification of the appropriate level for tests and full reporting of outcomes
☒	☐	Estimates of effect sizes (e.g. Cohen's *d*, Pearson's *r*), indicating how they were calculated

Our web collection on statistics for biologists contains articles on many of the points above.

Software and code

Policy information about availability of computer code

Data collection	Shimadzu LC-20AP, Agilent LC-MS 1260-6230, AB Sciex ZenoTOF 7600
Data analysis	GraphPad Prism version 9.1.2, SnapGene version 5.1.2, ImageJ version 1.8.0

For manuscripts utilizing custom algorithms or software that are central to the research but not yet described in published literature, software must be made available to editors and reviewers. We strongly encourage code deposition in a community repository (e.g. GitHub). See the Nature Portfolio guidelines for submitting code & software for further information.

Data

Policy information about availability of data

All manuscripts must include a data availability statement. This statement should provide the following information, where applicable:

- Accession codes, unique identifiers, or web links for publicly available datasets
- A description of any restrictions on data availability
- For clinical datasets or third party data, please ensure that the statement adheres to our policy

Streptomyces roseosporus genome sequence is available in GenBank with accession number GCA_023716005.1. The LimB homologs from Streptomyces coelicolor M145, Streptomyces albus J1074, and E. coli DH5α that were used in this study can be assessed at Uniprot (Q9X888, WP_0155508125.1, and WP_000130380.1). Materials including strains and plasmids are available upon reasonable request. Source data are provided with this manuscript.

Research involving human participants, their data, or biological material

Policy information about studies with human participants or human data. See also policy information about sex, gender (identity/presentation), and sexual orientation and race, ethnicity and racism.

Reporting on sex and gender	N/A
Reporting on race, ethnicity, or other socially relevant groupings	N/A
Population characteristics	N/A
Recruitment	N/A
Ethics oversight	N/A

Note that full information on the approval of the study protocol must also be provided in the manuscript.

Field-specific reporting

Please select the one below that is the best fit for your research. If you are not sure, read the appropriate sections before making your selection.

☒ Life sciences ☐ Behavioural & social sciences ☐ Ecological, evolutionary & environmental sciences

For a reference copy of the document with all sections, see nature.com/documents/nr-reporting-summary-flat.pdf

Life sciences study design

All studies must disclose on these points even when the disclosure is negative.

Sample size	Quantitative assays were performed in the at least three independent biological replicates and mean and standard deviation values calculated. The sample sizes were chosen based on previous publications in a similar field, considering what is necessary to generate a convincing and compelling result.
Data exclusions	No data were excluded from our manuscript.
Replication	In a parallel experiment, we set up triple replications for determining the mean value and standard deviation. All attempts at replication were successful. All the experiments were conducted at three times to verify the reproducibility.
Randomization	In a parallel experiment, we set up triple replications for determining the mean value and standard deviation. All attempts at replication were successful. All the experiments were conducted at three times to verify the reproducibility.
Blinding	Blinding is not relevant to our study. The data presented are quantitative values (obtained as described in Methods) and did not require subjective judgment or interpretation.

Reporting for specific materials, systems and methods

We require information from authors about some types of materials, experimental systems and methods used in many studies. Here, indicate whether each material, system or method listed is relevant to your study. If you are not sure if a list item applies to your research, read the appropriate section before selecting a response.

Materials & experimental systems

n/a	Involved in the study	
☐	☒	Antibodies
☒	☐	Eukaryotic cell lines
☒	☐	Palaeontology and archaeology
☒	☐	Animals and other organisms
☒	☐	Clinical data
☒	☐	Dual use research of concern
☒	☐	Plants

Methods

n/a	Involved in the study	
☒	☐	ChIP-seq
☒	☐	Flow cytometry
☒	☐	MRI-based neuroimaging

Antibodies

Antibodies used

Blinding is not relevant to our study. The data presented are quantitative values (obtained as described in Methods) and did not require subjective judgment or interpretation.

Validation

All antibodies were well-recognized clones in the field and validated by the manufactures. These antibodies are further validated and routinely used in our lab. Results in this study (Figure 1A, Figure 1B, Figure 3, Figure 5B, Figure 5C, Figure 6A, Figure 7C, and Supplementary Figure S1C, Figure S3, Figure S4B) also show that the antibodies work properly for this assay.

Plants

Seed stocks

N/A

Novel plant genotypes

N/A

Authentication

N/A
